# Genome-wide p63-Target Gene Analyses Reveal TAp63/NRF2-Dependent Oxidative Stress Responses

**DOI:** 10.1158/2767-9764.CRC-23-0358

**Published:** 2024-02-01

**Authors:** Marco Napoli, Avani A. Deshpande, Deepavali Chakravarti, Kimal Rajapakshe, Preethi H. Gunaratne, Cristian Coarfa, Elsa R. Flores

**Affiliations:** 1 Department of Molecular Oncology, Division of Basic Science, H. Lee Moffitt Cancer Center and Research Institute, Tampa, Florida.; 2 Cancer Biology and Evolution Program, H. Lee Moffitt Cancer Center and Research Institute, Tampa, Florida.; 3 Stellanova Therapeutics Inc., Houston, Texas.; 4 Sheikh Ahmed Center for Pancreatic Cancer Research, The University of Texas M.D. Anderson Cancer Center, Houston, Texas.; 5 Department of Biology and Biochemistry, University of Houston, Houston, Texas.; 6 Department of Molecular and Cellular Biology, Dan L Duncan Comprehensive Cancer Center, Baylor College of Medicine, Houston, Texas.

## Abstract

The p53 family member *TP63* encodes two sets of N-terminal isoforms, TAp63 and ΔNp63 isoforms. They each regulate diverse biological functions in epidermal morphogenesis and in cancer. In the skin, where their activities have been extensively characterized, TAp63 prevents premature aging by regulating the quiescence and genomic stability of stem cells required for wound healing and hair regeneration, while ΔNp63 controls maintenance and terminal differentiation of epidermal basal cells. This functional diversity is surprising given that these isoforms share a high degree of similarity, including an identical sequence for a DNA-binding domain. To understand the mechanisms of the transcriptional programs regulated by each p63 isoform and leading to diverse biological functions, we performed genome-wide analyses using p63 isoform-specific chromatin immunoprecipitation, RNA sequencing, and metabolomics of *TAp63^−^^/^^−^* and *ΔNp63^−^^/^^−^* mouse epidermal cells. Our data indicate that TAp63 and ΔNp63 physically and functionally interact with distinct transcription factors for the downstream regulation of their target genes, thus ultimately leading to the regulation of unique transcriptional programs and biological processes. Our findings unveil novel transcriptomes regulated by the p63 isoforms to control diverse biological functions, including the cooperation between TAp63 and NRF2 in the modulation of metabolic pathways and response to oxidative stress providing a mechanistic explanation for the *TAp63* knock out phenotypes.

**Significance:**

The p63 isoforms, TAp63 and ΔNp63, control epithelial morphogenesis and tumorigenesis through the interaction with distinct transcription factors and the subsequent regulation of unique transcriptional programs.

## Introduction

The p63 gene (*TP63*) is a member of the p53 family of transcription factors and is characterized by the presence of two alternative promoters, which drive the expression of two sets of isoforms: TAp63 and ΔNp63 (1). The former is characterized by the presence of an N-terminal acidic transactivation domain, resembling the transactivation domain of p53 (1). The latter lacks this domain, but it is still transcriptionally competent (2–4). In addition to the N-terminal regions, further complexity of the *TP63* gene is due to alternative splicing at the 3′ end of its mRNA, thus allowing for the generation of proteins with five different C-termini (α, β, γ, δ, and ε isoforms), with the α isoforms being the most abundant in epithelial tissues for both TAp63 and ΔNp63 (5, 6). The relevance of this variety in the p63 structure is underlined by the different functions fulfilled by these isoforms. Although the activities of the specific C-terminal portions are still under investigation, a growing body of evidence is available regarding the diverse biological functions associated with the two groups of N-terminal isoforms. The roles of *TP63* were first described in the skin due to the striking epidermal phenotypes of *TP63^−^^/^^−^* mice (7, 8) and to the syndromes observed in patients with *TP63* mutations, including ectodermal dysplasia, orofacial clefting, and limb malformations (9). TAp63 is the only p63 isoform detected in dermal stem cells, called skin precursor cells (SKP) and present within the dermal papilla and dermal sheath of the hair follicle, and it was shown to be essential for wound healing and hair regeneration (10, 11). TAp63 keeps SKPs in quiescence through transactivation of *Cdkn1c* (also known as *p57^KIP2^*), thus preventing premature depletion of these cells, early aging of the skin, alopecia, and wound healing defects (11). Indeed, *TAp63^−^^/−^* mice have a reduced lifespan and show accelerated signs of aging, including hair loss, impaired wound healing, and kyphosis (11). In line with this, epidermal cells derived from *TAp63^−^^/^^−^* mice at embryonic day 18.5 (E18.5) exhibit higher levels of genomic instability and senesce earlier than their wild-type counterparts (11). In addition, *TAp63^−^^/^^−^* mice have impaired glucose and lipid metabolism, ultimately leading to insulin resistance and liver steatosis (12). On the other hand, ∆Np63 is not expressed in SKPs and is generally restricted to the epidermal basal cells, where it controls their proliferation and terminal differentiation through the direct induction of the microprocessor complex subunit *Dgcr8* (3). Accordingly, *ΔNp63^−^^/^^−^* epidermal cells hyper proliferate, and do not differentiate; instead, they have the ability to self-renew and express factors associated with pluripotency, including Nanog, Oct4, and Sox2 (3). This leads to the severe phenotype of the *ΔNp63^−^^/^^−^* mice, which contain a disorganized layer of basal epidermal cells that fail to terminally differentiate (3).

In addition to exerting these diverse physiologic roles, TAp63 and ∆Np63 have opposite activities in tumor formation. TAp63 is a potent tumor and metastasis suppressor (13) and can regulate the expression of p53-regulated target genes, such as *Cdkn1a*, *Bax*, and *Lkb1* (12, 14, 15), as well as p53-independent genes, including both noncoding genes, such as a group of 9 long noncoding RNAs called *TROLLs* (16), as well as protein-coding genes, such as *Iapp*, *Smarcd3*, and *Dicer* (13, 17, 18). In fact, it is in part through induction of *Dicer* and the consequent impact on the miRNA biogenesis pathway that TAp63 suppresses tumor metastasis *in vivo* (13). On the contrary, ΔNp63 behaves as an oncogene by repressing the expression of p53 and TAp63 target genes (17, 19). In addition to being a transcriptional repressor, ΔNp63 is also a transcriptional activator, and can induce transcription of several p53- and TAp63-independent genes, as *T* (also known as *Brachyury*), *Jag1*, and *Dgcr8* (3, 4, 20), as well as some TAp63 targets, such as *Hagh* and *Perp* (21, 22).

Since the diverse *in vivo* roles of the p63 isoforms rely on their capacity to specifically transactivate different sets of genes, understanding how TAp63 and ΔNp63, which have identical DNA-binding domains, can regulate distinct transcriptional programs is of the utmost importance to understand the p63 biology and its implications in cancer. Here, we address this point by performing transcriptome profiling as well as detection of p63 isoform–specific binding sites. Using p63 isoform–specific knock out epidermal cells and chromatin immunoprecipitation coupled with next generation sequencing (ChIP-seq), we identified genes whose loci are bound and whose expression levels are affected by either or both p63 isoforms. In contrast to the strategies previously adopted by other groups that investigated the transcriptional activities of exogenously overexpressed p63 isoforms (23–28), we focused on the physiologic activity of the endogenous TAp63 and ΔNp63 proteins. Notably, the utilization of *TAp63^−^^/^^−^* and *ΔNp63^−^^/^^−^* mice provided us with the unique opportunity to analyze each isoform independently through the comparison of unstressed primary wild-type, *TAp63^−^^/^^−^* and *ΔNp63^−^^/^^−^* epidermal cells. Our findings demonstrate that the diversity between the TAp63- and the ΔNp63-regulated transcriptomes is due to the interaction and cooperation of these isoforms with distinct transcriptional factors, thus unveiling the mechanism employed by the p63 isoforms to regulate unique transcriptional programs and shedding new light on the p63 isoform–specific regulated biological processes. Finally, our results also reveal the crucial interaction between TAp63 and NRF2, the master regulator of the antioxidant response encoded by *Nfe2l2* (29), in controlling the expression of genes involved in the redox homeostasis and provide molecular insights to decipher the common defects displayed by both *TAp63^−^^/^^−^* and *Nfe2l2^−^^/^^−^* mice.

## Materials and Methods

### Cell Culture

Wild-type, *ΔNp63^−^^/^^−^*, and *TAp63^−^^/^^−^* epidermal cells were isolated from E18.5-day embryos as described previously (3, 11). Briefly, epidermal cells were isolated from skin by treatment with Dispase II (Roche). The separated epidermis was minced and incubated in 0.25% trypsin/EDTA (Gibco) for 20 minutes. Cells were plated on mitomycin c (Roche)-treated J2–3T3 feeder cells in F media (Sigma) supplemented with 0.4 mg/mL hydrocortisone, 24 ng/mL adenine, 8.4 ng/mL cholera toxin, 5 mg/mL insulin, 13 ng/mL 3,3,5-triiodo-L-thyronine, and 10 ng/mL EGF as described earlier (30). The hTERT immortalized human epidermal cells, KER-CT, were purchased from ATCC (Cat# CRL-4048, RRID:CVCL_S877) and grown in KBM-Gold BulletKit media (Lonza). All the cell lines were maintained as mycoplasma negative.

### Chromatin Immunoprecipitation

Wild-type, *ΔNp63^−^^/^^−^*, and *TAp63^−^^/^^−^* epidermal cells were grown to near confluence on J2–3T3 feeder cells in F media as described previously (3, 11). Feeder cells were removed with 0.02% EDTA 24 hours prior to collecting epidermal cells for chromatin extraction. Cellular proteins were crosslinked to DNA using 1% formaldehyde and chromatin was prepared as described earlier (3, 11). Each chromatin immunoprecipitation (ChIP) was performed in triplicate using epidermal cells from three embryos of each genotype. The p63 bound DNA to be sequenced was immunoprecipitated using the H129 p63 antibody (sc-8344, Santa Cruz Biotechnology, RRID:AB_653766). For the validation experiments, the utilized antibodies were: ΔNp63 (sc-8609, Santa Cruz Biotechnology, RRID:AB_10612539) and TAp63 (sc-8608, Santa Cruz Biotechnology, RRID:AB_2207181). For re-ChIP experiments, the complexes from each sample of the primary ChIP assays were eluted in 50 µL of 10 mmol/L dithiothreitol (DTT) for 30 minutes at 37°C, before being diluted in dilution buffer (1% SDS, 10 mmol/L EDTA, 50 mmol/L Tris HCl pH 8.1) and incubated with the following antibodies: Foxj2 (sc-514265, Santa Cruz Biotechnology, RRID:AB_2941799), Foxl1 (ab190226, Abcam, RRID:AB_2941798), Nrf2 (#12721, Cell Signaling Technology, RRID:AB_2715528), Stat4 (#2653, Cell Signaling Technology, RRID:AB_2255156), and Stat5a (#9363, Cell Signaling Technology, RRID:AB_2196923). As a negative control for the immunoprecipitation, IgGs purified from mouse serum (sc-2025, Santa Cruz Biotechnology, RRID:AB_737182) and rabbit serum (sc-2027, Santa Cruz Biotechnology, RRID:AB_737197) were used. The recruitment of the different transcription factors was analyzed by qRT-PCR. Primers for the analysis of the p63 bound chromatin regions are listed in Supplementary Table S1.

### ChIP Sequencing Analysis

Each of the chromatin immunoprecipitation sequencing (ChIP-seq) samples yielded 38–42 million raw reads 75 nt long; the reads were mapped onto the mouse genome UCSC mm10 (Genome Reference Consortium GRCm38) using BOWTIE2 (RRID:SCR_005476; ref. 31). High-resolution genome-wide maps were derived using the BEDTOOLS software (RRID:SCR_006646; ref. 32) and visualized in UCSC Genome Browser (RRID:SCR_005780). Finally, the MACS2 peak calling package (RRID:SCR_013291; ref. 33) was used to identify enriched regions using default parameters. Normalized sequence tag density around peaks was computed using the HOMER software (RRID:SCR_010881; ref. 34) and plotted using the Python language scientific library. Peak distribution across genomic domains including Upstream 10 kb, 5′UTR, Exon, 3′UTR, Intron, and Downstream 10 kb was evaluated using the BEDTOOLS software and the GENCODE gene model (RRID:SCR_014966; ref. 35). Motif analysis was performed using MEME-ChIP (RRID:SCR_001783; ref. 36) with default parameters, and plotted using the Weblogo software (RRID:SCR_010236; ref. 37). Nrf2 ChIP-seq datasets in epithelial cells were downloaded from GEO accession numbers GSE87357 (38) and GSE122504 (39) and processed as described above. Union of the Nrf2 peaks from the two datasets was computed and used to distinguish Nrf2 motifs versus non-Nrf2 motifs. Conservation of peak sets between mouse mm10 and human genome hg38 was assessed using the liftOver software (RID: SCR_018160; ref. 40). Venn diagrams between different peak sets were determined using the BEDTOOLS software (32).

### Identification of the p63 Isoform–Specific Cofactors

An in-house software was used to screen a collection of validated peaks against the collection of transcription factor motifs compiled by the Molecular Signature Database (RRID:SCR_016863; ref. 41).

### RNA-Seq and Analysis

Approximately 5 µg of polyA+ RNA was used to construct RNA-seq libraries using the standard Illumina protocol. Mouse sequencing yielded 30–40 million read pairs for each sample. The mouse mRNA-seq reads were mapped using Hisat2 (RRID:SCR_015530; ref. 42) onto the mouse genome build UCSC mm10 (Genome Reference Consortium GRCm38). Gene expression was quantified using featureCounts (43) and the GENCODE gene model (35). Gene expression differences were computed using the RUVSeq (RRID:SCR_006263; ref. 44) and Deseq2 (RRID:SCR_015687; ref. 45) R statistical system packages, with statistical significance achieved for FDR-adjusted *P* < 0.05 and fold change exceeding 1.25x. Principal component analysis (PCA) was executed using the implementation within the R statistical analysis system. Hierarchical clustering of samples was executed by first computing the symmetrical sample distance matrix using the Pearson correlation between mRNA profiles as a metric, then using the R statistical system. Heat maps were generated using the Python language scientific library. For gene signatures, we further explored gene enrichment using hypergeometric distribution as well as Gene Set Enrichment Analysis (GSEA) and the gene set compendium compiled by the Molecular Signature Database (MSigDB; ref. 41).

### Integration of RNA-Seq and ChIP-Seq Data

Gene targets associated with peak on mm10 were determined using the BEDTOOLS software (32) with a 10-kb window around genes, using the GENCODE gene model (35), and are listed in Supplementary Table S2. Differentially enriched pathways were determined using the hypergeometric distribution, with significance achieved for FDR-adjusted *P* < 0.05, and are listed in Supplementary Table S3.

### Quantitative Real-Time PCR

Total RNA was prepared using TRIzol reagent (Invitrogen; ref. 13). For gene expression analysis, complementary DNA was synthesized from 5 µg of total RNA using the SuperScript II First-Strand Synthesis Kit (Invitrogen) according to the manufacturer's protocol followed by qRT-PCR using the SYBR Fast qPCR master mix (Kapa Biosystems). qRT-PCR was performed using a QuantStudio 6 Flex (Applied Biosystems) Real-time PCR machine. Each qRT-PCR was performed in triplicate using epidermal cells from three embryos of each genotype. Primers for mouse *ΔNp63*, *TAp63*, and *GAPDH* were used as described previously (3, 11). The sequences of the additionally utilized primers are listed in Supplementary Table S4.

### Protein Interactions and Western Blot Analysis

Wild-type, *ΔNp63^−^^/^^−^*, and *TAp63^−^^/^^−^* epidermal cells were grown to near confluence on J2–3T3 feeder cells, which were removed with 0.02% EDTA 24 hours prior to collecting the epidermal cells for the immunoprecipitation of endogenous ΔNp63 and TAp63. The harvested keratinocytes were lysed in 50 mmol/L Tris-HCl pH 8.0, 100 mmol/L NaCl, 1 mmol/L MgCl2, 1 mmol/L EDTA, 1% Triton X-100, 10% glycerol supplemented with phosphatase and protease cocktail inhibitors (Roche), and lysates were cleared by centrifugation. Lysates were precleared with protein G-agarose (Millipore) for 30 minutes before incubation for 2 hours with either ΔNp63 (sc-8609, Santa Cruz Biotechnology, RRID:AB_10612539) or TAp63 (sc-8608, Santa Cruz Biotechnology, RRID:AB_2207181) antibody. As a negative control for the immunoprecipitation, IgGs purified from rabbit serum (sc-2027, Santa Cruz Biotechnology, RRID:AB_2207181) were used. Beads were washed three times in 50 mmol/L Tris-HCl pH 7.5, 150 mmol/L NaCl, 1 mmol/L EDTA, 0.1% NP-40. The immunocomplexes were then electrophoresed on a 10% SDS PAGE and transferred to polyvinylidene difluoride membrane. Blots were probed with primary antibodies for ΔNp63 (1:1,000; #619002, BioLegend, RRID:AB_2207170), TAp63 (1:500; #938102, BioLegend, RRID:AB_2876758), Foxj2 (1:500; sc-514265, Santa Cruz Biotechnology, RRID:AB_2941799), Foxl1 (1:1,000; PA5–40518, Invitrogen, RRID:AB_2607990), Nrf2 (1:500; PA5–27882, Invitrogen, RRID:AB_2545358), Stat4 (1:1,000; sc-398228, Santa Cruz Biotechnology, RRID:AB_2810272), and Stat5a (1:1,000; 13–3600, Invitrogen, RRID:AB_2533013), at 4°C for 18 hours followed by incubation for 1 hour at room temperature with the appropriate secondary antibodies conjugated to horseradish peroxidase (1:5,000; Jackson Laboratory). Detection was performed using the ECL Plus Kit (Amersham) following the manufacturer's protocol and X-ray autoradiography.

### siRNA Transfection

1 × 10^6^ cells per 10-cm dishes were transfected with 40 nmol/L Mission siRNA oligonucleotides (Mission siRNA, Sigma) using jetPRIME (Polyplus) according to the manufacturer's protocol. The universal negative control #1 (siControl; Sigma) was used as negative control. The siRNA to target human TAp63 was previously reported (17). To target the remaining mRNAs of interest, pools of three siRNAs were used, whose sequences are listed in Supplementary Table S5.

### Nontargeted Metabolite Profiling

Wild-type and *TAp63^−^^/^^−^* epidermal cells were grown to near confluence on J2–3T3 feeder cells, which were removed with 0.02% EDTA 24 hours prior to collecting the epidermal cells. The epidermal cells were quickly washed in cold PBS and extracted in 80% methanol. The extracts were cleared by centrifugation, and the metabolites in the supernatant were analyzed by liquid chromatography-high resolution mass spectrometry (LC-HRMS). The identified metabolites are listed in Supplementary Table S6 and were then analyzed via the MetaboAnalyst software (RRID: SCR_015539) to identify the differentially enriched pathways.

### Reactive Oxygen Species Measurement

Reactive oxygen species (ROS) were measured using the CellROX green reagent (Invitrogen) according to the manufacturer's instructions. In short, KER-CT cells transfected with the indicated siRNAs were plated in 6-well plates at 70% confluence. The next day, cells were treated with 31.25 µmol/L H_2_O_2_ for 1 hour at 37 °C. CellROX green reagent was then added to a final concentration of 5 µmol/L, and the cells were incubated for additional 30 minutes at 37°C. After three washes with PBS, the cells were then kept in Live Cell Imaging Solution (Invitrogen) while pictures were acquired with a Zeiss Observer.Z1 microscope. The CellRox mean fluorescence intensity was then quantified with ImageJ and represented as fold changes versus untreated KER-CT cells transfected with the negative control siRNA.

### Data Availability Statement

The ChIP-seq data were deposited to NCBI Gene Expression Omnibus (GEO) repository (series GSE147723). All other data in this article can be obtained from the corresponding authors upon reasonable request.

## Results

### Endogenous ∆Np63 and TAp63 Bind to Distinct Regions Throughout the Genome

**FIGURE 1:**
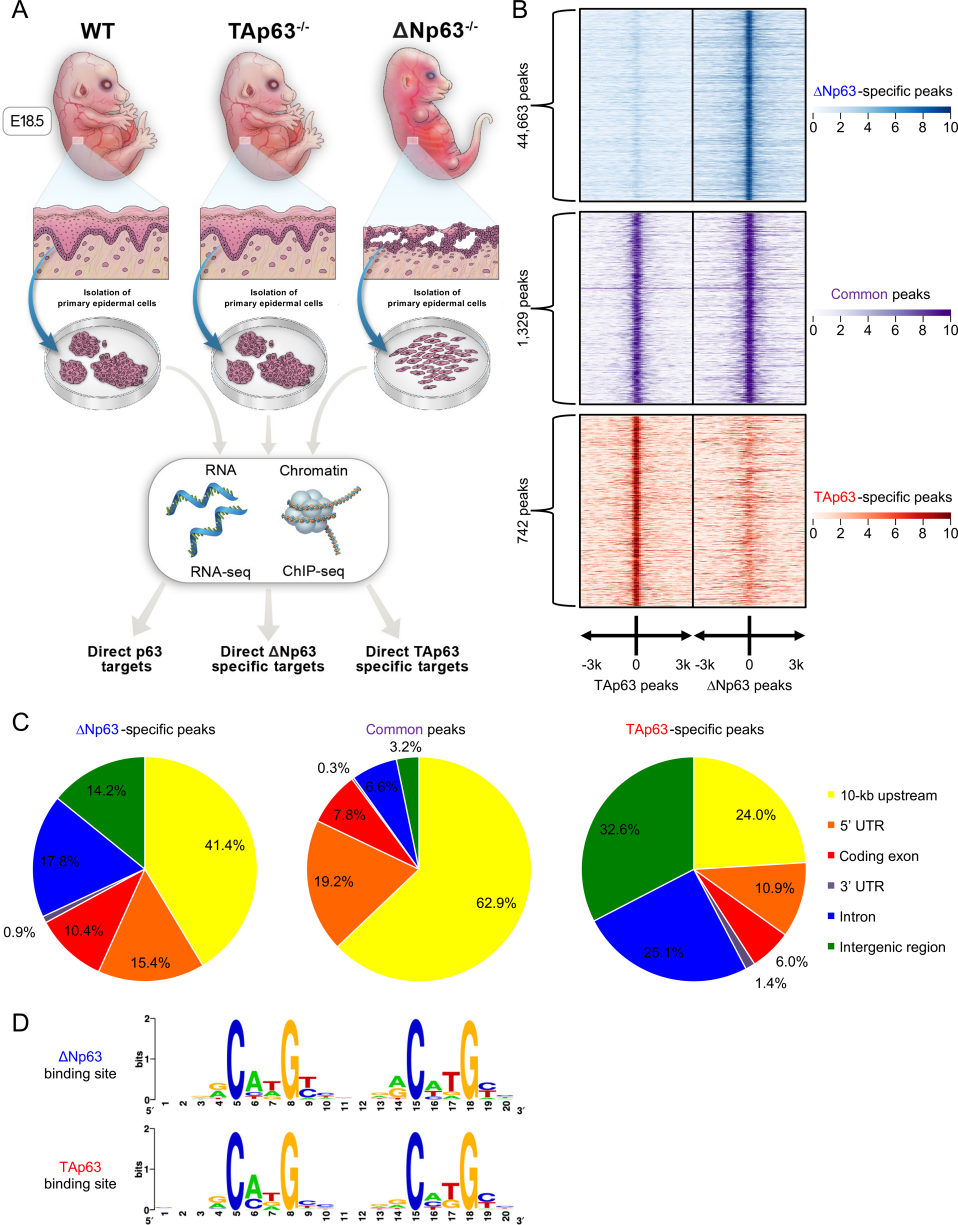
Endogenous ∆Np63 and TAp63 bind to distinct regions throughout the genome. **A,** Experimental outline. WT, *ΔNp63^−^^/^^−^*, and *TAp63^−^^/^^−^* epidermal cells were isolated from embryonic day 18.5 (E18.5) embryos. RNA extracted from these cells was used for expression profiling by RNA-seq, and chromatin was used for genome-wide analysis of p63 binding to DNA by ChIP-seq. The overlap in genes identified from these analyses revealed: (i) direct p63 target genes; (ii) direct ΔNp63-specific target genes; and (iii) direct TAp63-specific target genes. **B,** Table summarizing the total number of peaks found through the ChIP-sequencing analysis and the relative p63 isoform responsible for the binding. **C,** Distribution of the ΔNp63-specific (left), common (center), and TAp63-specific (right) ChIP-seq peaks with respect to genomic landmarks. **D,** Nucleotide distribution in the ∆Np63-specific (top) and TAp63-specific (bottom) DNA response elements identified in the ChIP-seq peaks.

Several groups including ours have previously identified a number of transcriptional target genes uniquely regulated by ∆Np63 (3, 4) or TAp63 (12, 13, 16, 17); yet, mechanisms used by these isoforms to achieve target selectivity remain unknown. To determine the mechanisms defining selective transcriptional targets and DNA binding by each p63 isoform, we analyzed epidermal cells derived from *ΔNp63^−^^/^^−^*, *TAp63^−^^/^^−^,* and wild-type mice. In these cells, the expression of each p63 isoform is not altered by the absence of the other one (Supplementary Fig. S1A–S1C; refs. 3, 11). Epidermal cells have widely been used to analyze p63 functions, due to the crucial role of this transcription factor in the formation and maintenance of skin (46) and of other epithelial tissues (47–50). Indeed, p63 isoform–specific knockout epidermal cells represent a model of the respective phenotypes of the *ΔNp63^−^^/^^−^* and *TAp63^−^^/^^−^* mice. *ΔNp63^−^^/^^−^* epidermal cells hyperproliferate, self-renew, and fail to terminally differentiate similarly to the poorly differentiated epidermis of *ΔNp63^−^^/^^−^* mice (3), whereas *TAp63^−^^/^^−^* epidermal cells are hypoproliferative and senesce prematurely as *TAp63^−^^/^^−^* mice undergo depletion of SKPs and premature aging (11). As depicted in Fig. 1A, these epidermal cells with distinct phenotypes were then utilized to obtain the genome-wide spectra of genes bound and regulated by either ΔNp63, TAp63, or both by performing ChIP-seq and RNA-seq analyses. To ensure the immunoprecipitation of both p63 isoforms in the same conditions and therefore allowing for the comparison of the corresponding bound genomic regions, an antibody directed against the α isoforms, the predominant TAp63 and ΔNp63 isoforms in epithelial tissues (5, 6), was utilized. The ChIP-seq data were then mapped on the mouse genome (UCSC mm10) and peaks were defined using MACS2 (33). Among the identified peaks, the number of peaks exclusively bound by TAp63 was lower compared with either the common peaks or those exclusively bound by ΔNp63 (Fig. 1B). This is presumably due to the fact that TAp63 is expressed at low levels until it is induced in response to stress signals, such as wound healing, UV irradiation, or metabolic stress (5, 11, 12, 51), and it is similar to what has been reported for p53 ChIP-seq performed in untreated/unstressed cells of both mouse (52) and human origin (53). Next, we analyzed the genomic location of these peaks with respect to annotated genes. While almost two thirds of the common peaks were localized within 10-kb upstream of known genes, the isoform-specific peaks were more evenly distributed with the top 3 categories of the ΔNp63-specific peaks being 10-kb upstream of known genes, intron, and 5′ UTR, whereas the top three categories of the TAp63-specific peaks were intergenic regions, intron, and 10-kb upstream of known genes (Fig. 1C). To verify whether the recruitment of ∆Np63 and TAp63 to distinct genomic regions was due to a difference in these DNA sequences, we analyzed these sequences using MEME-ChIP software (36) to identify the ∆Np63- and TAp63-binding sites (Fig. 1D). As confirmed by a Pearson correlation analysis (PCA) (Supplementary Fig. S1D), these two motifs are highly similar, thus ruling out that in physiologic conditions endogenous ΔNp63 and TAp63 bind to distinct response elements. Taken together, our data indicate that the endogenous p63 isoforms can bind to different genomic DNA regions for gene regulation, even though this is not due to the recognition of distinct DNA response elements.

### Identification of ΔNp63 and TAp63 Regulated Transcriptomes

**FIGURE 2:**
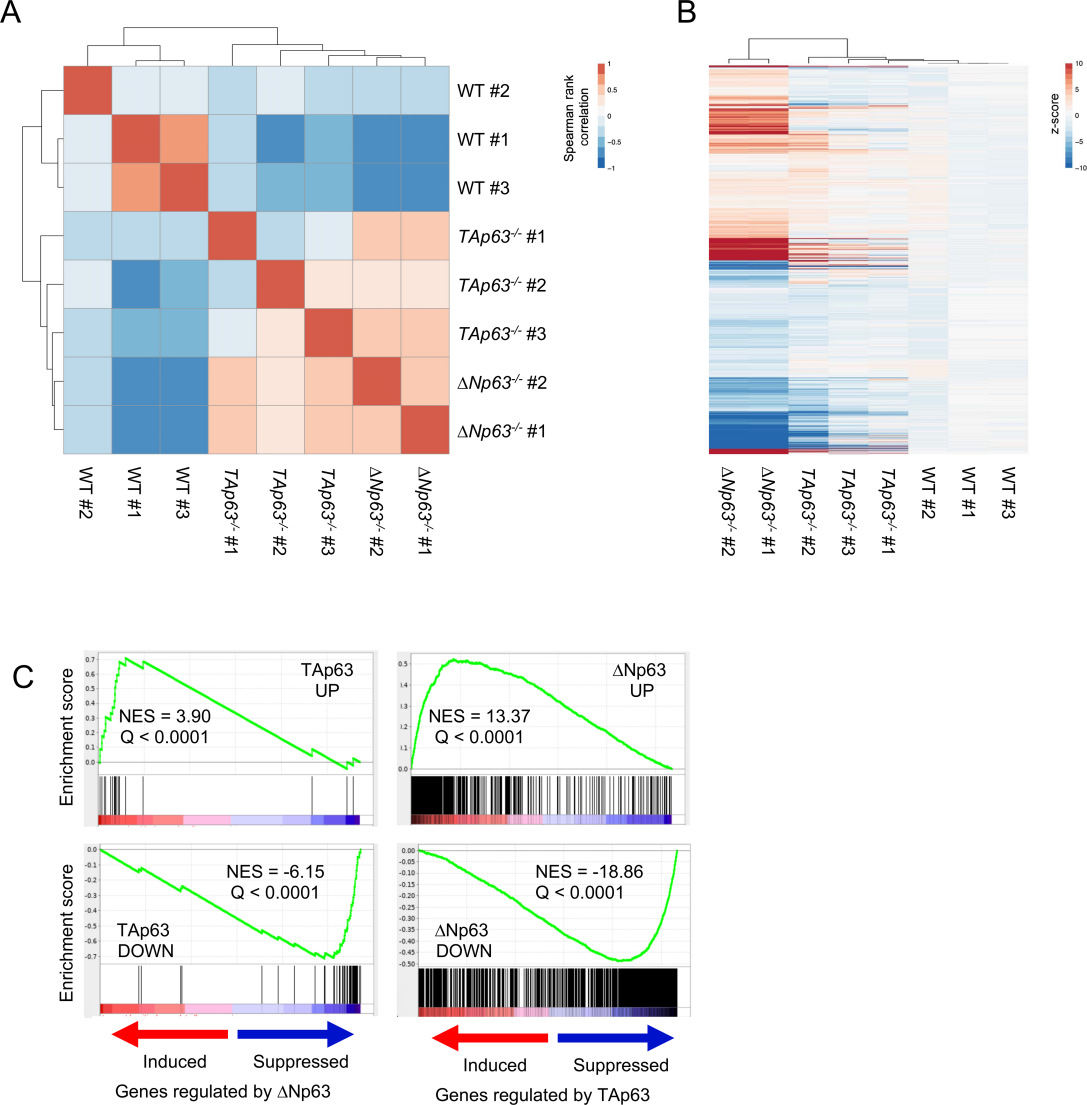
Identification of ∆Np63 and TAp63 regulated transcriptomes. **A,** Unsupervised clustering of the transcriptional profiles in WT, *ΔNp63^−^^/^^−^*, and *TAp63^−^^/^^−^* epidermal cells using Pearson correlation coefficient (PCC). **B,** Heat map visualization of the genes affected in either *ΔNp63^−^^/^^−^* or *TAp63^−^^/^^−^* epidermal cells. **C,** Reciprocal gene set enrichment analysis (GSEA) of the genes induced or repressed by the indicated p63 isoform (FC 8x, FDR < 0.05).

To identify genes whose expression is regulated by endogenous ∆Np63 and TAp63 in epidermal cells, we used a genome-wide approach by performing RNA-seq from the same set of epidermal cells utilized for the ChIP-seq analysis. Pearson's correlation coefficient-based clustering of the samples showed that they grouped according to the respective genotype (Fig. 2A). Compared with the gene expression levels detected in the wild-type epidermal cells, deletion of *ΔNp63* or *TAp63* caused variation in the expression of specific subsets of genes. Specifically, our RNA-seq data showed that 2,607 were differentially expressed in *TAp63^−^^/^^−^* versus WT epidermal cells and 9,146 genes were differentially expressed in *∆Np63^−^^/^^−^* versus WT epidermal cells. Of these genes, 2,114 genes were differentially expressed in both cases (Fig. 2B; Supplementary Fig. S2A–S2E). To globally analyze the gene sets differentially regulated by ∆Np63 and TAp63, we conducted gene-set enrichment analysis (GSEA; ref. 41) separately against the genes upregulated and downregulated by each isoform. Even though the genes with the greatest up- or downregulation by TAp63 or ∆Np63 were reciprocally enriched (Fig. 2C, Q < 0.0001), most of the genes differentially expressed compared with wild-type epidermal cells were generally affected in the same direction (836 upregulated and 1,086 downregulated genes), while only a minority of common regulated genes were expressed in the opposite manner (111 genes downregulated by ∆Np63 and upregulated by TAp63, and 81 genes upregulated by ∆Np63 and downregulated by TAp63, Supplementary Fig. S2A–S2E). Overall, these findings indicate that endogenous ∆Np63 and TAp63 regulate unique sets of target genes in epidermal cells.

### ∆Np63 and TAp63 Control Diverse Biological Processes by Cooperating With Distinct Transcription Factors

**FIGURE 3:**
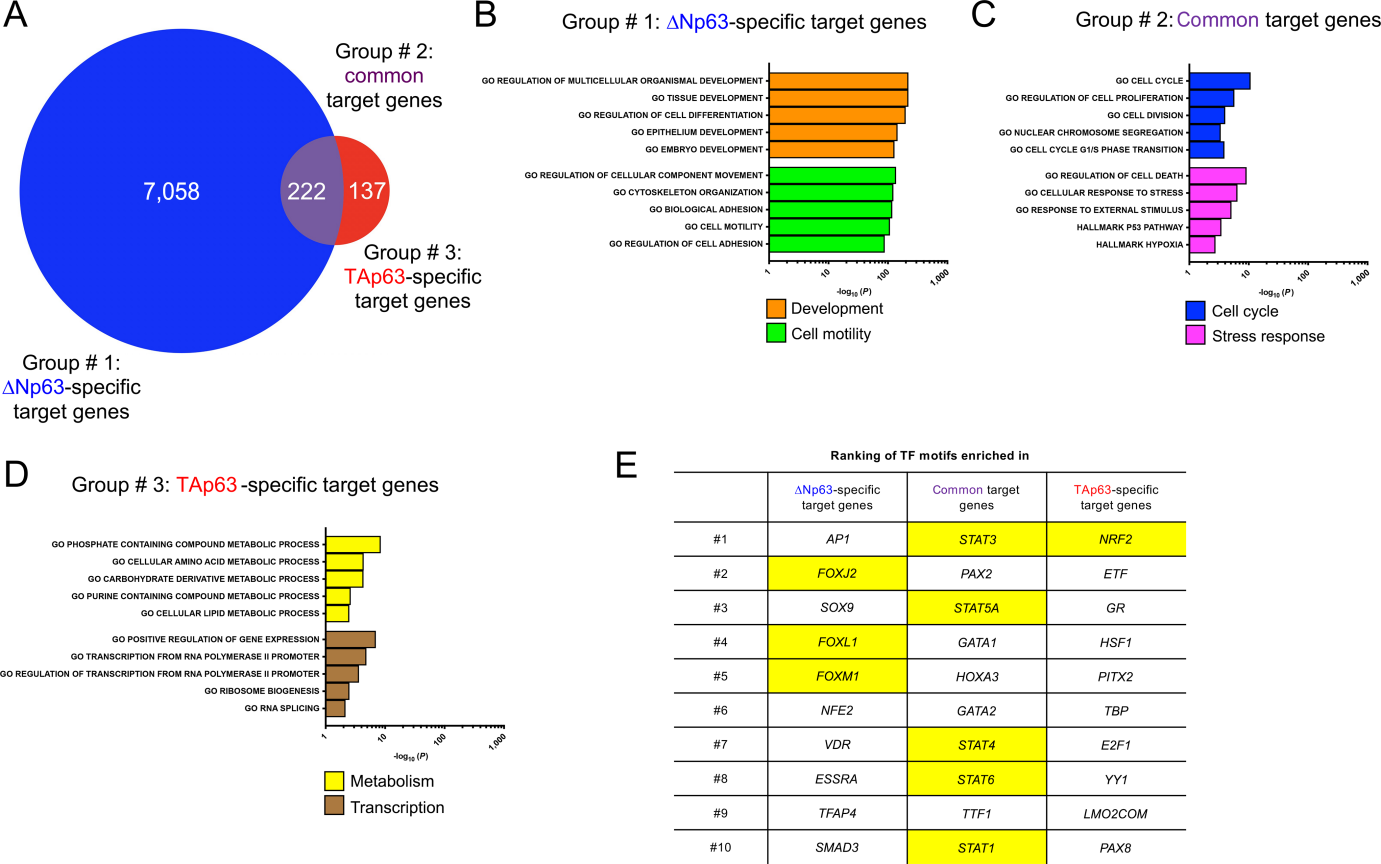
∆Np63 and TAp63 control distinct biological processes by cooperating with different transcription factors. **A,** Venn diagrams of ΔNp63-specific, common, and TAp63-specific direct target genes, with p63 ChIP-seq peaks ± 10 kb from their genomic loci. **B–D,** List of the differentially enriched pathways in ΔNp63-specific, common, and TAp63-specific direct target genes. **E,** Table listing the top 10 differentially enriched motifs in the peaks ± 10 kb from ΔNp63-specific, common, and TAp63-specific direct target genes. Highlighted in yellow are the motifs of the transcription factors selected for validation.

To exclude any indirect transcriptional effects associated to the deletion of either p63 isoform (e.g., upregulation of Nanog, Oct4, and Sox2 in *∆Np63^−^^/^^−^* epidermal cells; ref. 3), we focused specifically on genes that were directly affected by ∆Np63 and TAp63 either in a positive or negative manner by selecting targets whose genomic locus has a maximal distance of 10 kb from a peak identified through our ChIP-seq data and whose expression levels significantly changed based on our RNA-seq data. These criteria allowed us to select potential direct target genes, which were further divided into three groups: (i) 7,058 genes regulated only by ΔNp63; (ii) 222 genes regulated by both ΔNp63 and TAp63; and (iii) 137 genes regulated only by TAp63 (Fig. 3A; Supplementary Table S2). The identification of differentially enriched pathways using over-representation analysis (FDR-adjusted *P* < 0.05) showed that these three different transcriptional programs are involved in distinct biological processes associated with the p63 isoforms, confirming the validity of using epidermal cells to investigate the functions of ΔNp63 and TAp63 (Supplementary Table S3). Indeed, in the ∆Np63-specific group there was an enrichment for pathways involved in development (46) and cell motility (ref. 6; Fig. 3B). Regarding the pathways enriched in the common target genes, we identified pathways with established links with both ∆Np63 and TAp63, such as cell-cycle regulation (54) and stress response (ref. 55; Fig. 3C). Finally, in the case of the TAp63-specific group, we found enrichment for pathways involved in metabolism (12) and transcription (ref. 56; Fig. 3D).

Because the unique activities of ΔNp63 and TAp63 rely on the respective ability to modulate distinct gene sets, and given that gene expression is a process finely tuned by transcriptional factors acting in concert with other components, such as additional transcription factors and epigenetic modifiers (57), we evaluated the possibility that the specificity of ∆Np63 and TAp63 in regulating their target genes could be due to the cooperation with different factors. To address this, we focused our attention on the 2-kb regions surrounding the p63 peaks in each of the 3 groups of genes (i.e., ∆Np63-specific, common, and TAp63-specific target genes), and surveyed them for the differential abundance for any of the over 600 transcription factor motifs present in the Molecular Signatures Database (41). Notably, in each group we found preferentially present motifs for transcription factors whose functions are involved in the pathways associated with the respective p63 isoform (Supplementary Table S7). Indeed, peaks present in the ∆Np63-specific target genes were found to be enriched for members of the Forkhead box (Fox) family of transcription factors (Fig. 3E), known to be crucial regulators of development and epithelial to mesenchymal transition (58). For peaks of target genes regulated by both p63 isoforms, there was an enrichment for members of the Stat family of transcription factors (Fig. 3E), whose signaling affects cell survival and response to stress cues (59). Finally, in the peaks bound by TAp63 to modulate its target genes, the most enriched motif was that of Nrf2 (also known as Nfe2l2; Fig. 3E), a master regulator of multiple metabolic pathways (29).

### ∆Np63 Cooperates With the FOX Family Members to Regulate Cell Motility Genes

**FIGURE 4:**
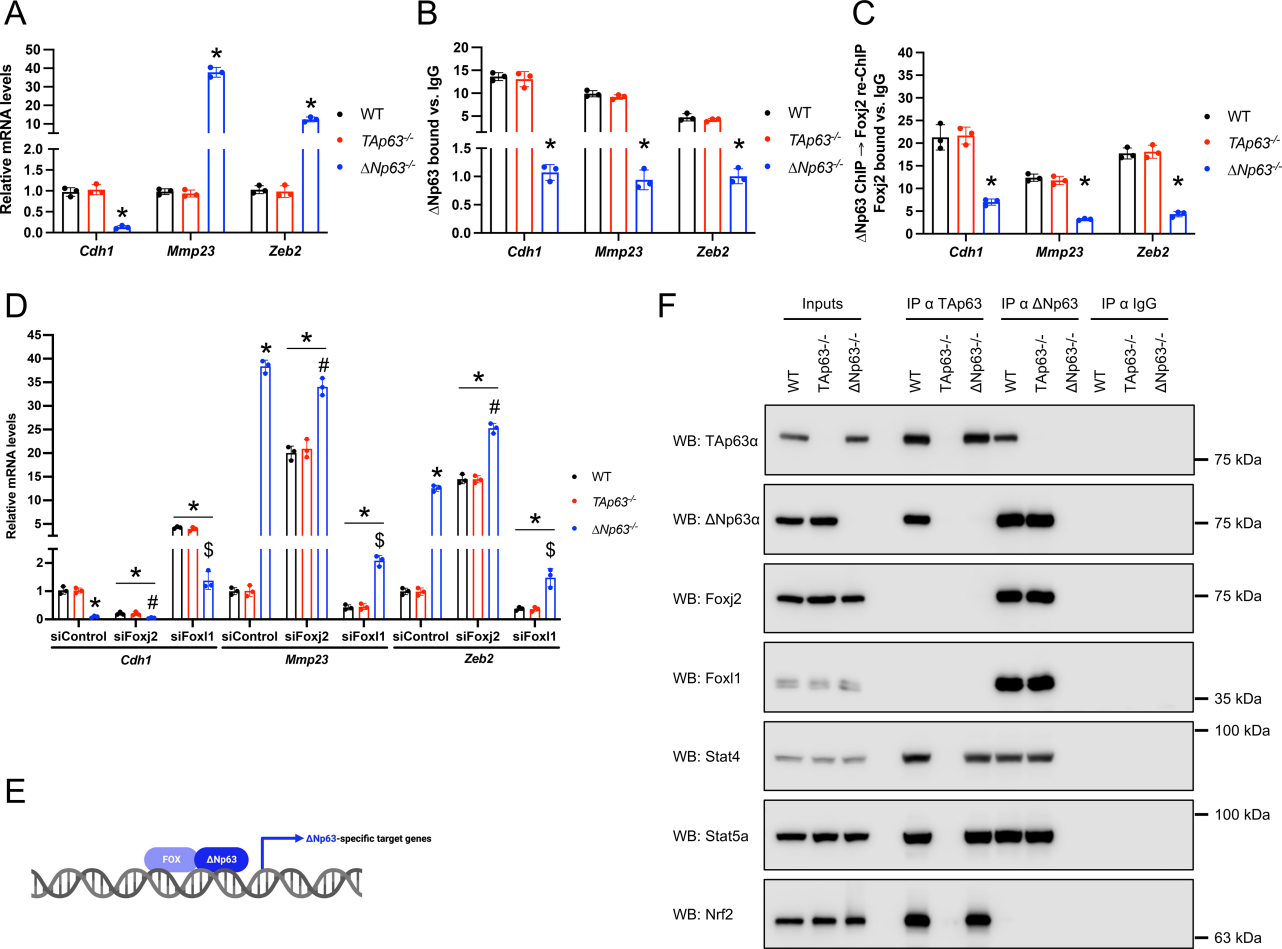
∆Np63 cooperates with the FOX family members to regulate cell motility genes. **A,** qRT-PCR for the indicated ΔNp63-specific target genes in epidermal cells of the indicated genotype. Data are mean ± SD, *n* = 3, * versus WT, *P <* 0.005, two-tailed *t* test. **B,** qRT-PCR of ∆Np63 ChIP assays using epidermal cells of the indicated genotype on the ∆Np63-specific peaks of the indicated ΔNp63-specific target genes. Data are mean ± SD, *n* = 3, * versus WT, *P <* 0.005, two-tailed *t* test. **C,** qRT-PCR of Foxj2 ChIP-re-ChIP assays using the ∆Np63 chromatin immunoprecipitated genomic regions of the indicated ΔNp63-specific target genes from epidermal cells of the indicated genotype. Data are mean ± SD, *n* = 3. * versus WT, *P <* 0.005, two-tailed *t* test. **D,** qRT-PCR of the indicated ΔNp63-specific target genes in WT, *ΔNp63^−^^/^^−^*, and *TAp63^−^^/^^−^* epidermal cells transfected with the indicated siRNAs. Data are mean ± SD, *n* = 3, * versus WT siControl, ^#^ versus WT siFoxj2, and ^$^ versus WT siFoxl1, *P* < 0.005, two-tailed *t* test. **E,** Model depicting the coordinated regulation of ∆Np63-specific cell motility genes by ∆Np63 and the FOX family members, FoxJ2 and Foxl1. **F,** Representative Western blot analysis using the indicated antibodies of the endogenous interacting proteins immunoprecipitated with p63 isoform–specific antibodies. IgGs from normal rabbit serum were used as negative control. Protein inputs (5% of lysates) are also shown.

To confirm the cooperation between the p63 isoforms and the transcription factors identified via our motif analysis, from each of the three groups of target genes, we selected three genes that were not only regulated by the respective p63 isoform but whose peaks also included the motif for the identified transcription factors. In the case of the ∆Np63-specific target genes, we selected three cell motility–associated genes (*Cdh1*, *Mmp23*, and *Zeb2*) whose peaks included motifs for the proepithelial factor Foxj2 and the promesenchymal factor Foxl1. We first confirmed that ∆Np63, but not TAp63, controlled their expression (Fig. 4A) and directly bound to their loci (Fig. 4B; Supplementary Fig. S3A), thus confirming that these genes are exclusively and directly regulated by ΔNp63. To assess for the possible binding of Foxj2 and Foxl1 to these chromatin regions bound by ∆Np63, ChIP assays for ∆Np63 followed by re-ChIP assays for either Foxj2 or Foxl1 were performed. Notably, we found that both Foxj2 and Foxl1 were recruited to these three genomic regions in a ∆Np63-dependent manner (Fig. 3C; Supplementary S3B), thus suggesting a coregulation of these ΔNp63 target genes. To verify whether Foxj2 and Foxl1 are required for the ΔNp63-dependent regulation of *Cdh1*, *Mmp23*, and *Zeb2*, we performed ChIP assays for ΔNp63 after depletion of either Fox family member. In these conditions, the binding of ΔNp63 to these 3 promoters was affected in a statistically significant manner (Supplementary Fig. S3C) and, in line with that, we observed a change in the expression of these 3 genes (Fig. 4D), indicating that the ability of ΔNp63 to regulate these targets is, at least in part, dependent on Foxj2 and Foxl1(Fig. 3E). This is further supported by immunoprecipitation assays performed in wild type, *TAp63^−^^/^^−^,* and *ΔNp63^−^^/^^−^* primary epidermal cells, showing that Foxj2 and Foxl1 physically interact with ΔNp63 but not with TAp63 (Fig. 4F). Notably, the downregulation of the transcription factors found to be enriched in the case of either common (Stat4 and Sta5a) or TAp63-specific genes (Nrf2) did not affect the expression of these ΔNp63-specific target genes (Supplementary Fig. S3D). Taken together, these data indicate that ΔNp63 works in concert with the FOX family members, Foxj2 and Foxl1, to regulate the expression of genes involved in cell motility.

### ∆Np63 and TAp63 Control the Expression of their Common Target Genes Together With Stat Proteins

**FIGURE 5:**
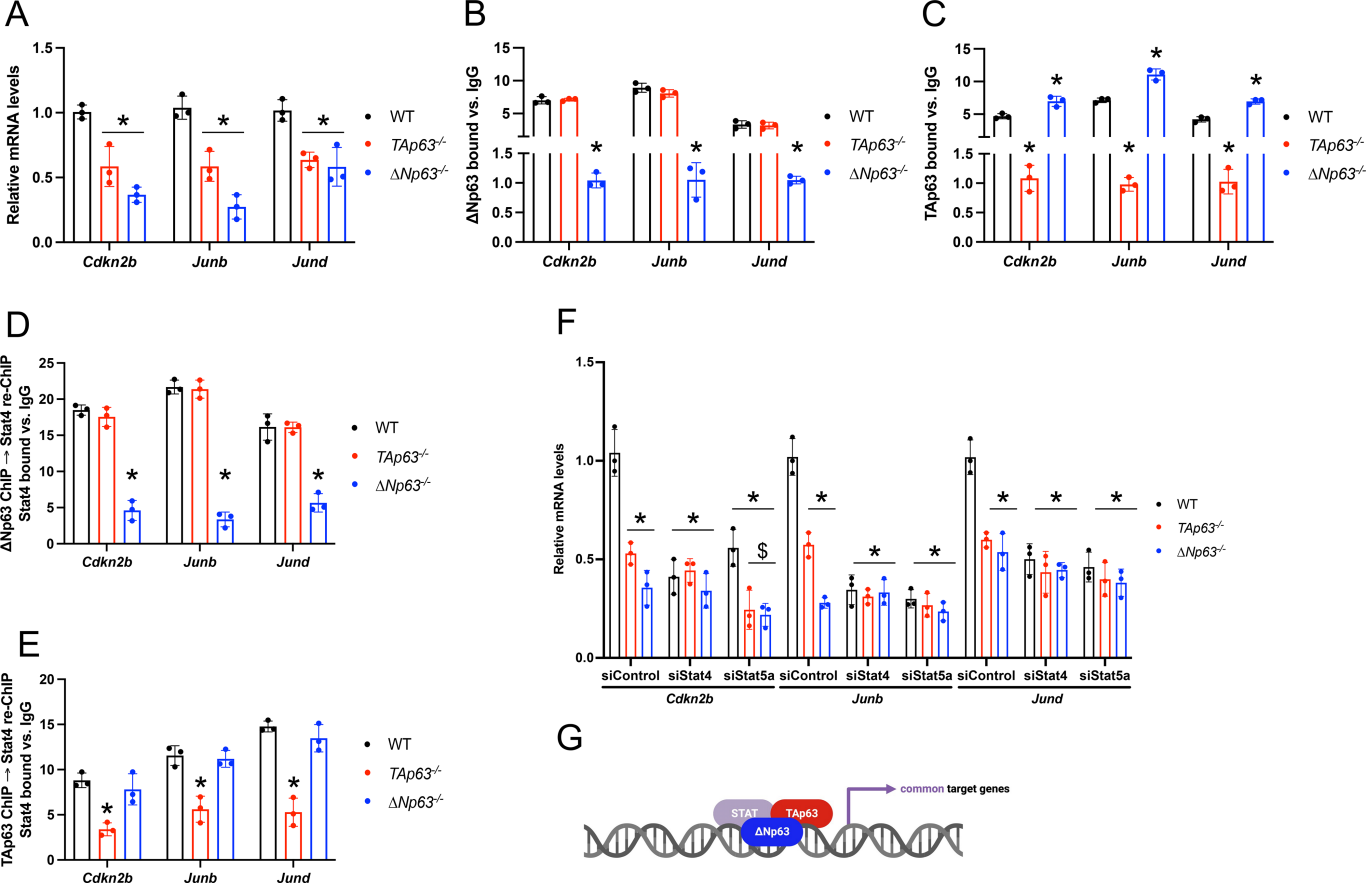
∆Np63 and TAp63 control the expression of their common target genes together with Stat proteins. **A,** qRT-PCR for the indicated ΔNp63 and TAp63 common target genes in epidermal cells of the indicated genotype. Data are mean ± SD, *n* = 3, * versus WT, *P <* 0.005, two-tailed *t* test. **B,** qRT-PCR of ∆Np63 ChIP assays using epidermal cells of the indicated genotype on the common peaks of the indicated common target genes. Data are mean ± SD, *n* = 3, * versus WT, *P <* 0.005, two-tailed *t* test. **C,** qRT-PCR of TAp63 ChIP assays using epidermal cells of the indicated genotype on the same genomic regions as in B. Data are mean ± SD, *n* = 3, * versus WT, *P <* 0.005, two-tailed *t* test. **D,** qRT-PCR of Stat4 ChIP-re-ChIP assays using the ∆Np63 chromatin immunoprecipitated genomic regions of the indicated common target genes from epidermal cells of the indicated genotype. Data are mean ± SD, *n* = 3. * versus WT, *P <* 0.005, two-tailed *t* test. **E,** qRT-PCR of Stat4 ChIP-re-ChIP assays using the TAp63 chromatin immunoprecipitated genomic regions of the indicated common target genes from epidermal cells of the indicated genotype. Data are mean ± SD, *n* = 3. * versus WT, *P <* 0.005, two-tailed *t* test. **F,** qRT-PCR of the indicated common target genes in WT, *TAp63^−^^/^^−^*, and *ΔNp63^−^^/^^−^* epidermal cells transfected with the indicated siRNAs. Data are mean ± SD, *n* = 3, * versus WT siControl, ^#^ versus WT siStat4, and ^$^ versus WT siStat5a, *P* < 0.005, two-tailed *t* test. **G,** Model depicting the coordinated regulation of ΔNp63 and TAp63 common target genes with the Stat family members, Stat4 and Stat5a.

To investigate the transcriptional mechanism used by ∆Np63 and TAp63 for the regulation of their common target genes, we considered the target genes regulated by both p63 isoforms and selected three genes (*Cdkn2*, *Junb*, and *Jund*), which are involved in cell-cycle regulation and stress response and whose p63-bound chromatic regions have motifs for the Stat family members, Stat4 or Stat5a. Both ∆Np63 and TAp63 affected the expression of these 3 genes (Fig. 5A) and were found to bind to their genomic loci (Fig. 5B and C), indicating that indeed this gene set is controlled by both p63 isoforms. It is worth noting that the binding of TAp63 was higher in *ΔNp63^−^^/^^−^* cells (Fig. 5C), suggesting that the presence of ΔNp63 could inhibit the proper recruitment of TAp63 to the commonly controlled promoters, in line with previous observations (17, 60) as well as with our immunoprecipitation data showing that TAp63 and ΔNp63 interact in wild-type keratinocytes (Fig. 4F). Next, we verified whether TAp63 and ∆Np63 could cooperate with Stat4 and Stat5a in regulating their common target genes. We performed ChIP assays for either TAp63 or ∆Np63, whose eluates were utilized to perform re-ChIP assays for either Stat4 (Fig. 5D and E) or Stat5a (Supplementary Fig. S4A and S4B). Importantly, we found that both Stat family members are recruited to the same genomic regions of *Cdkn2*, *Junb*, and *Jund* bound by TAp63 and ∆Np63 (Fig. 5D and E; Supplementary S4A and S4B). Downregulation of either *Stat4* or *Stat5a* impaired the recruitment of both TAp63 and ∆Np63 on these genomic regions (Supplementary Fig. S4C and S4D) and affected the expression of *Cdkn2*, *Junb*, and *Jund* (Fig. 5F), indicating that TAp63 and ∆Np63 regulate these genes by binding cooperatively with Stat4 and Stat5a (Fig. 5G). Accordingly, both p63 isoforms were found to interact with these two Stat family members in immunoprecipitation assays (Fig. 4F). In contrast, downregulation of the transcription factors found to be enriched in the case of either ΔNp63-specific (Foxj2 and Foxl1) or TAp63-specific genes (Nrf2) did not affect the expression of these common target genes (Supplementary Fig. S4E). All together, these findings indicate that ∆Np63 and TAp63 regulate their common target genes involved in cell-cycle regulation and stress response together with the Stat family members, Stat4 and Stat5a.

### TAp63 and NRF2 Coordinately Regulate the Expression of TAp63-Specific Target Genes

**FIGURE 6:**
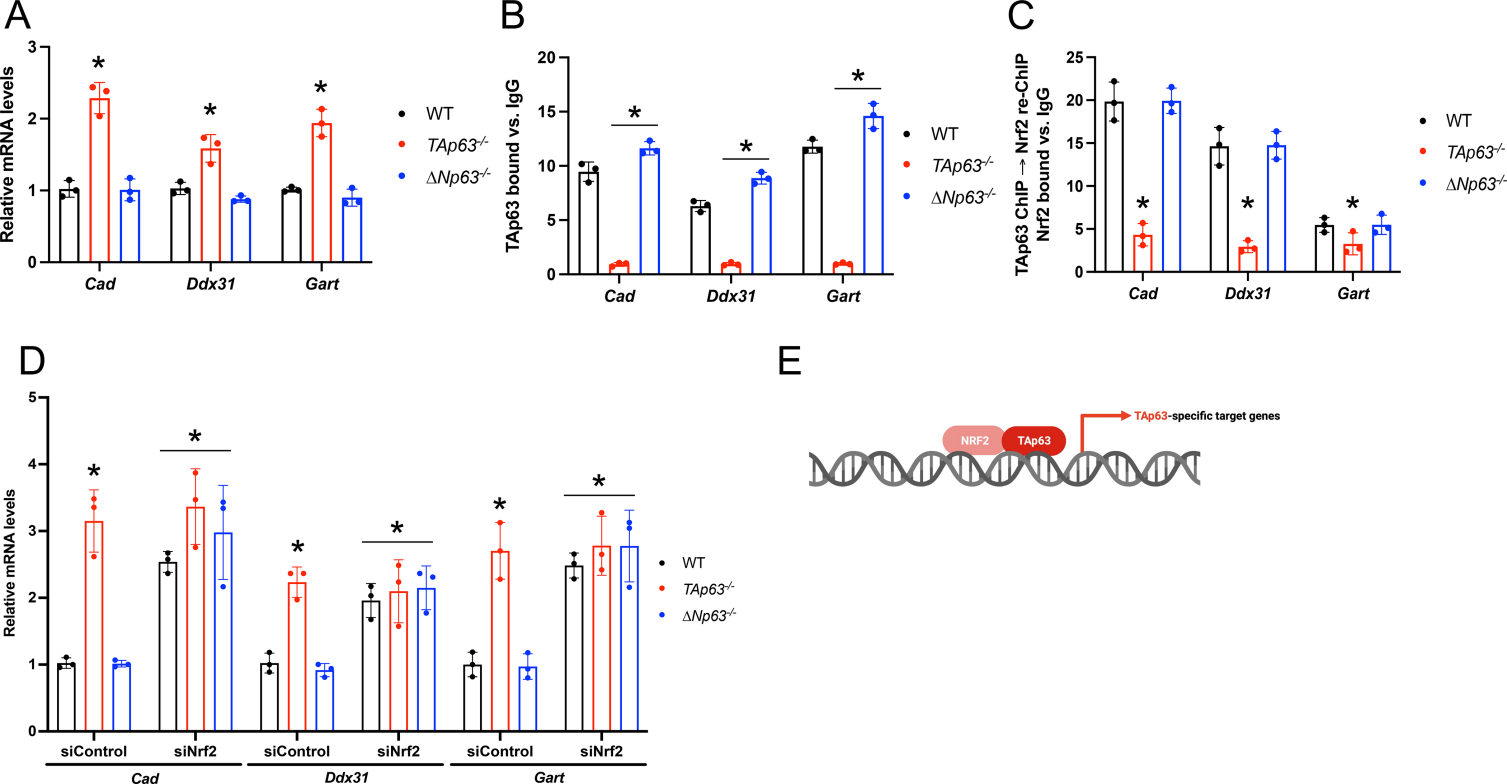
TAp63 and NRF2 coordinately regulate the expression of TAp63-specific target genes. **A,** qRT-PCR for the indicated TAp63-specific target genes in epidermal cells of the indicated genotype. Data are mean ± SD, *n* = 3, * versus WT, *P <* 0.005, two-tailed *t* test. **B,** qRT-PCR of TAp63 ChIP assays using epidermal cells of the indicated genotype on the TAp63-specific peaks of the indicated TAp63-specific target genes. Data are mean ± SD, *n* = 3, * versus WT, *P <* 0.005, two-tailed *t* test. **C,** qRT-PCR of Nrf2 ChIP-re-ChIP assays using the TAp63 chromatin immunoprecipitated genomic regions of the indicated TAp63-specific target genes from epidermal cells of the indicated genotype. Data are mean ± SD, *n* = 3. * versus WT, *P <* 0.005, two-tailed *t* test. **D,** qRT-PCR of the indicated TAp63-specific target genes in WT, *ΔNp63^−^^/^^−^*, and *TAp63^−^^/^^−^* epidermal cells transfected with the indicated siRNAs. Data are mean ± SD, *n* = 3, * vs. WT siControl, ^#^ versus WT siNrf2, *P* < 0.005, two-tailed *t* test. **E,** Model depicting the coordinated regulation of TAp63-specific metabolic genes by TAp63 and NRF2.

To determine how TAp63 regulates the expression of its unique transcriptional program, we focused on genes involved in metabolism and transcriptional regulation and selected three genes (*Cad*, *Ddx31*, *Gart*) whose TAp63-bound chromatin regions also included motifs for the antioxidant transcription factor Nrf2. First, we confirmed that TAp63, but not ∆Np63, regulates the expression of these three genes (Fig. 6A) and directly bound to their loci (Fig. 6B and Supplementary Fig. S5A), indicating that these genes are exclusively and directly regulated by TAp63. We then assessed for the involvement of Nrf2 in the regulation of these genes by performing ChIP assays for TAp63 followed by re-ChIP assays for Nrf2. Notably, we found that the binding of Nrf2 was impaired in the *TAp63^−^^/^^−^* epidermal cells (Fig. 6C), indicating that Nrf2 regulates these target genes at least in part in a TAp63-dependent manner. Conversely, we performed ChIP assays for TAp63 in *Nrf2* knockdown cells and observed an impairment in TAp63 recruitment to these three genomic regions (Supplementary Fig. S5B), which ultimately led to a change in the expression levels of *Cad*, *Ddx31*, *Gart* (Fig. 6D). The cooperation between TAp63 and Nrf2 was also confirmed via immunoprecipitation assays performed in wild-type, *TAp63^−^^/^^−^,* and *ΔNp63^−^^/^^−^* primary epidermal cells, showing that Nrf2 physically interacts with TAp63 but not with ΔNp63 (Fig. 4F). On the other hand, downregulation of the transcription factors found to be enriched in the case of either ΔNp63-specific (*Foxj2* and *Foxl1*) or common target genes (*Stat4* and *Sta5a*) did not affect the expression of these TAp63-specific target genes (Supplementary Fig. S5C). Taken together, these data indicate that TAp63 controls the expression of its specific target genes involved in metabolism and transcriptional regulation by interacting with Nrf2 to activate TAp63/Nrf2-specific targets.

### The p63 Isoform–specific Biological Processes are Maintained Across Multiple Epithelial Tissues

**FIGURE 7:**
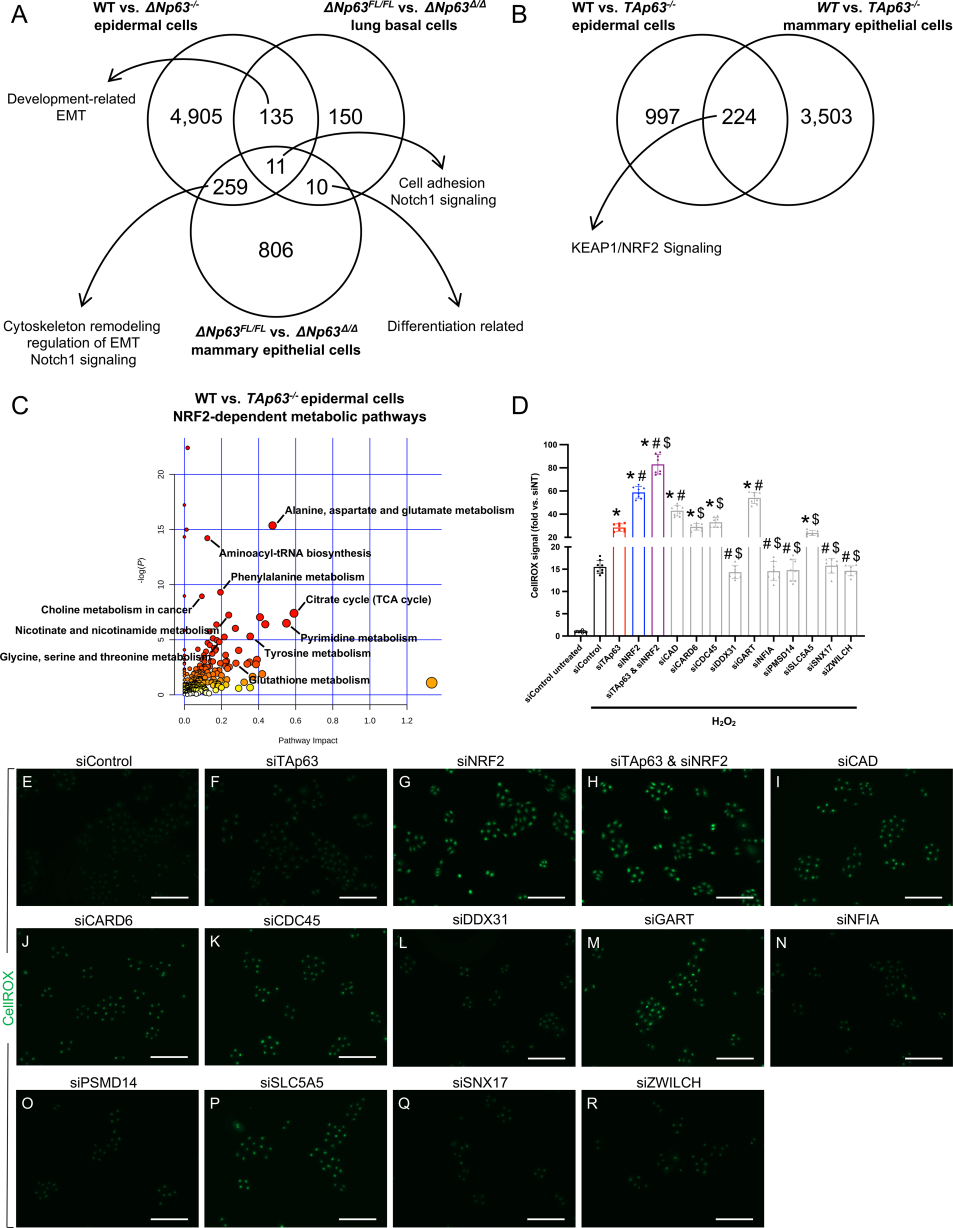
TAp63 promotes the NRF2-dependent oxidative stress response. **A,** Venn diagram of the differentially regulated genes in the indicated *ΔNp63*-competent versus deleted murine primary epithelial cell types. Listed are the pathways enriched in each respective section. **B,** Venn diagram of the differentially regulated genes in the indicated *TAp63*-competent versus deleted murine primary cell types. Listed are the pathways enriched in both epithelial cell types. **C,** Scatter plot of the metabolic pathways differentially regulated in WT versus *TAp63^−^^/^^−^* epidermal cells. Labeled are the NRF2-dependent metabolic pathways. **D,** Quantification of the CellRox signal in KER-CT cells transfected with the indicated siRNAs and treated with H_2_O_2_. The CellRox signal is normalized with respect to the siControl untreated cells. Data are mean ± SD, *n* = 8, * versus siControl H_2_O_2_, ^#^ versus siTAp63 H_2_O_2_, and ^$^ versus siNRF2 H_2_O_2_, *P* < 0.005, two-tailed *t* test. **E–R,** Representative images of CellRox signal in KER-CT cells transfected with the indicated siRNAs and treated with H_2_O_2_. Scale bars, 100 µm.

To evaluate whether our findings in mouse primary epidermal cells may be indicative of the p63 isoform–specific activities also in other epithelial tissues, we compared the transcriptional programs and relative pathways regulated by either ∆Np63 or TAp63 in epidermal cells with RNA-seq datasets from other mouse epithelial tissues previously reported by our group, including lung basal cells and mammary epithelial cells. For ∆Np63, we used our gene expression profiles obtained in *ΔNp63*-competent versus depleted lung basal cells (61) and mammary epithelial cells (62). Despite most of the individual genes regulated by ∆Np63 were cell type specific, there was a commonality at the pathway level, including development and differentiation related pathways as well as cell adhesion, cytoskeleton remodeling, and regulation of epithelial to mesenchymal transition (Fig. 7A; Supplementary Table S8), indicating a common ∆Np63 activity across these various epithelial cell types. In the case of TAp63, we compared our RNA-seq from WT and *TAp63^−^^/^^−^* epidermal cells with the RNA-seq from WT and *TAp63^−^^/^^−^* mammary epithelial cells (62), and among the genes regulated by TAp63 in both epithelial cell types, there were genes involved in the KEAP1/NRF2 pathway, including *Nqo1* and *Txnrd1* (Fig. 7B; Supplementary Table S8). These data therefore indicate that the unique biological processes regulated by each p63 isoform are performed across multiple epithelial cell types.

### TAp63 Promotes the NRF2-Dependent Oxidative Stress Response

The regulation of canonical Nrf2 target genes by TAp63 identified via our comparison across epithelial tissues is noteworthy, because it may underlie the commonalities shared by mouse models lacking either of these transcription factors, such as reduced fatty acid oxidation, increased fatty acid synthesis, insulin resistance, and liver steatosis (12, 63, 64). Prompted by our findings, we tested whether TAp63 may affect metabolic processes known to be controlled by Nrf2 by performing a nontargeted metabolite profiling of WT and *TAp63^−^^/^^−^* epidermal cells via liquid chromatography-high resolution mass spectrometry (LC-HRMS) (Supplementary Table S6). Importantly, when we analyzed the identified metabolites via the MetaboAnalyst software to look for differentially enriched pathways, we found that lack of *TAp63* affected multiple metabolic pathways known to be regulated by Nrf2, including glutathione metabolism, nicotinate, and nicotinamide metabolism, as well as glycine, serine, and threonine metabolism (Fig. 7C). Given the relevance of these Nrf2-dependent pathways in controlling redox homeostasis (29), we assessed whether TAp63 and Nrf2 may cooperate in regulating redox metabolism via a set of commonly regulated genes. To do this, we compared our list of TAp63-regulated genes associated to peaks including an Nrf2 motif with publicly available Nrf2 ChIP-seq datasets (38, 39). By evaluating the conservation of these common mouse genes regulated by TAp63 and Nrf2 in the human genome, we identified the following group of 10 genes: *CAD*, *CARD6*, *CDC45*, *DDX31*, *GART*, *NFIA*, *PSMD14*, *SLC5A5*, *SNX17*, and *ZWILCH*. Next, we tested the requirement of any of these 10 genes in response to oxidative stress in human immortalized KER-CT cells. After treatment with H_2_O_2_, we detected a 15-fold increase in the amount of reactive oxygen species (ROS) in the control transfected cells (Fig. 7D). Importantly, lack of either *TAp63* or *NRF2* further enhanced ROS levels, with the downregulation of both factors achieving an 80-fold increase compared with the untreated siControl-transfected cells (Fig. 7D). Notably, downregulation of 5 of these 10 TAp63/NRF2 regulated genes (*CAD*, *CARD6*, *CDC45*, *GART*, and *SLC5A5*) caused an increase in ROS levels, indicating their relevance in redox metabolism (Fig. 7D–R). Importantly, two of them, *CAD* and *GART*, caused an increase in ROS levels even in untreated KER-CT cells, similar to the downregulation of *NRF2* (Supplementary Fig. S6A–S6O). It is worth noting that in untreated KER-CT cells, downregulation of both *TAp63* and *NRF2* caused the highest increase in ROS levels, underlying their cooperation in modulating redox homeostasis in physiologic conditions (Supplementary Fig. S6A–S6O). Taken together, our results have unveiled a novel cooperation between TAp63 and NRF2 in regulating redox homeostasis and the response to oxidative stress via the modulation of a common set of target genes conserved in the mouse and human genomes.

## Discussion

The *TP63* gene belongs to the p53 family of transcription factors and expresses two different sets of isoforms: TAp63 and ΔNp63 (1). Even though they share a high level of identity, except for an acidic portion present exclusively at the N-terminus of TAp63 (1), these two p63 isoforms exert distinct functions both in physiologic and pathologic conditions as highlighted by the different phenotypes of *ΔNp63^−^^/^^−^* and *TAp63^−^^/^^−^* mice. *ΔNp63* is crucial for the terminal differentiation of the epidermis (3), while its overexpression endows cancer cells with oncogenic properties in several human cancer types (17, 65–67). In contrast, the absence of *TAp63* causes premature aging associated with skin blisters, ulcerations, and alopecia, the inability to maintain stem cells required for wound healing and hair regeneration (SKPs) in quiescence (11), and the spontaneous onset of highly metastatic tumors (13, 16, 62). Because the biological processes affected by the p63 isoforms are controlled through their unique transcriptional activities, understanding how TAp63 and ΔNp63 regulate distinct transcriptional programs – despite having an identical DNA binding domain – is essential to characterize their dichotomous behaviors. Here, we report our genome-wide approach using ChIP-seq coupled with RNA-seq conducted in primary epidermal cells derived from p63 isoform–specific knock out mice (*ΔNp63^−^^/^^−^* and *TAp63^−^^/^^−^*). By taking advantage of these unique mouse models and cellular reagents, we found that TAp63 and ΔNp63 regulate distinct transcriptional programs associated with biological processes in line with the unique phenotypes of the p63 isoforms, thus underlying the validity of our experimental strategy. Our integrated approach, including bioinformatic analyses, ChIP-re-ChIP assays and immunoprecipitation experiments, allowed us to demonstrate that ΔNp63 directly controls the expression of genes involved in development and cell motility by binding to and working together with Foxj2 and Foxl1, while TAp63 acts in concert with NRF2 to regulate the expression of genes important for metabolic pathways and transcriptional regulation. Conversely, the genes affected by both p63 isoforms are associated with cell-cycle control and response to stress cues and are regulated via the cooperation with the Stat family members, Stat4 and Stat5a. Notably, our findings are not limited to epidermal cells. Indeed, we found that, despite the difference observed at the individual gene level, the unique biological processes controlled by ΔNp63 and TAp63 are maintained across other primary epithelial cell types, including lung basal cells (61) and mammary epithelial cells (62).

Because our analysis of the chromatin regions exclusively bound by either ΔNp63 or TAp63 did not reveal significant differences in their respective DNA-binding sites, we hypothesized that the different transcriptional activities of the p63 isoforms may rely on their ability to interact and cooperate with different transcription factors. Accordingly, when we focused our attention on the p63 peaks associated with the 3 distinct groups of genes (i.e., ΔNp63-specific, common, and TAp63-specific target genes), we found that these DNA sequences were differentially enriched for distinct transcription factor motifs. These results together with metabolomics analysis and cross epithelial RNA-seq experiments allowed us to discover a previously unrecognized collaboration between TAp63 and NRF2. Indeed, we found that they act in concert to balance the redox homeostasis and that their concurrent downregulation further enhanced the levels of reactive oxygen species (ROS) detected when either of them is silenced. This effect relies on the ability of TAp63 and NRF2 to bind to each other and to control the expression of a common set of 10 genes, whose regulatory sites are conserved between the mouse and the human genomes. Not only did several of these genes affected ROS levels when downregulated in H_2_O_2_-treated human immortalized epidermal cells, but notably two of them, *CAD*, which encodes for an enzyme involved in the *de novo* synthesis of pyrimidines (68), and *GART*, which encodes for an enzyme involved in the *de novo* synthesis of purines (69), also affected ROS levels in untreated cells, indicating their crucial role as TAp63/NRF2 target genes in modulating redox balance and further explaining the similarities in the functions of these two genes (12, 63, 64).

For the ΔNp63-specific transcriptional program, we found that ΔNp63 regulates some of its target genes by interacting and working in concert with Foxj2 and Foxl1, two members of the Forkhead box family. They are pivotal regulators of cytoskeleton remodeling and epithelial to mesenchymal transition, as well as differentiation related pathways (70, 71). In addition, their overexpression is oncogenic in multiple human cancers (72), similar to the effects due to the aberrant expression of ΔNp63 (17, 65–67). Importantly, when ΔNp63 regulates target genes that are also controlled by TAp63, these p63-bound genomic regions contain motifs for different transcription factors, including multiple members of the Stat family. Given the well-established role of the Stat signaling in cell-cycle regulation and cell death (73, 74), we selected for validation genes affected by both p63 isoforms and involved in these biological processes. Importantly, we found that both TAp63 and ΔNp63 physically and functionally interact with Stat4 and Stat5a and are recruited to the same genomic regions to coordinately tune the expression of genes involved in cell-cycle control and stress response.

Taken together, our data provide novel mechanistic insights to the long-standing question in the p63 field on how TAp63 and ΔNp63 transcriptionally control very different biological processes in multiple epithelial cell types even though these p63 isoforms share the same DNA-binding domain. Indeed, our findings demonstrate that the distinct transcriptional programs and pathways regulated by TAp63 and ΔNp63 are determined by the cooperative binding of each isoform with specific transcription factors, a specificity possibly relying on the diverse N-terminal domains of TAp63 and ΔNp63. These results provide a new mechanistic understanding of the function of TAp63 and ΔNp63 and how their distinct downstream transcriptomes are regulated, thus impacting different biological processes in the skin and other epithelial tissues, and in cancer where p63 isoforms are expressed at different levels. Moreover, our data reveal novel p63 biology and open new avenues to explore for therapeutic intervention of epidermal diseases and cancer therapy.

## Supplementary Material

Supplementary Figure S1 Endogenous ∆Np63 and TAp63 bind to distinct regions throughout the genome

Supplementary Figure S2 Identification of ∆Np63 and TAp63 regulated transcriptomes

Supplementary Figure S3 ∆Np63 cooperates with the FOX family members to regulate cell motility genes

Supplementary Figure S4 ∆Np63 and TAp63 control the expression of their common target genes together with Stat proteins

Supplementary Figure S5 TAp63 and NRF2 coordinately regulate the expression of TAp63-specific target genes

Supplementary Figure S6 TAp63 promotes the NRF2-dependent oxidative stress response

Table S1 List of DNA oligonucleotides for the TAp63 and ∆Np63 ChIP assays

Table S2 List of genes differentially expressed between of TAp63-/- or ΔNp63-/- mouse epidermal cells and the WT mouse epidermal cells. For each gene, the count of the TAp63 or ∆Np63 bound peaks within 10 kb from the gene bodies is indicated.

Table S3 List of biological pathways differentially enriched (FDR-adjusted P < 0.01) in TAp63-/- and/or ΔNp63-/- mouse epidermal cells versus the WT mouse epidermal cells. The pathways are grouped based on the biological processes and the differentially expressed genes for each pathways are also listed.

Table S4 List of DNA oligonucleotides to assess the expression levels of the TAp63 and ∆Np63 target genes.

Table S5 List of the siRNA sequences.

Table S6 List of identified metabolites detected via liquid chromatography-high resolution mass spectrometry in TAp63-/- vs. WT mouse epidermal cells.

Table S7 List of transcriptional factor motifs differentially enriched in the 3 groups of TAp63-specific direct target genes, ∆Np63-specific direct target genes, and common direct target genes. For the analysis of the transcription factor motifs only the 2 kb regions surrounding the p63 peaks in each of the 3 groups of genes were considered.

Table S8 List of differentially expressed genes in ΔNp63+/+ vs. ΔNp63-/- cells (mouse epidermal, lung basal, and mammary epithelial cells) or in TAp63+/+ vs. TAp63-/- cells (mouse epidermal and mammary epithelial cells).
