# Supplementary material for: Genome-wide p63-Target Gene Analyses Reveal TAp63/NRF2-Dependent Oxidative Stress Responses
Source: Cancer Res Commun. 2024 Feb 1;4(2):264–78. doi: 10.1158/2767-9764.CRC-23-0358 (PMC10832605; doi:10.1158/2767-9764.CRC-23-0358)
Supplement: Supplementary Figure S1 — Endogenous ∆Np63 and TAp63 bind to distinct regions throughout the genome [file crc-23-0358-s01.pdf]

Supplementary Figure 1

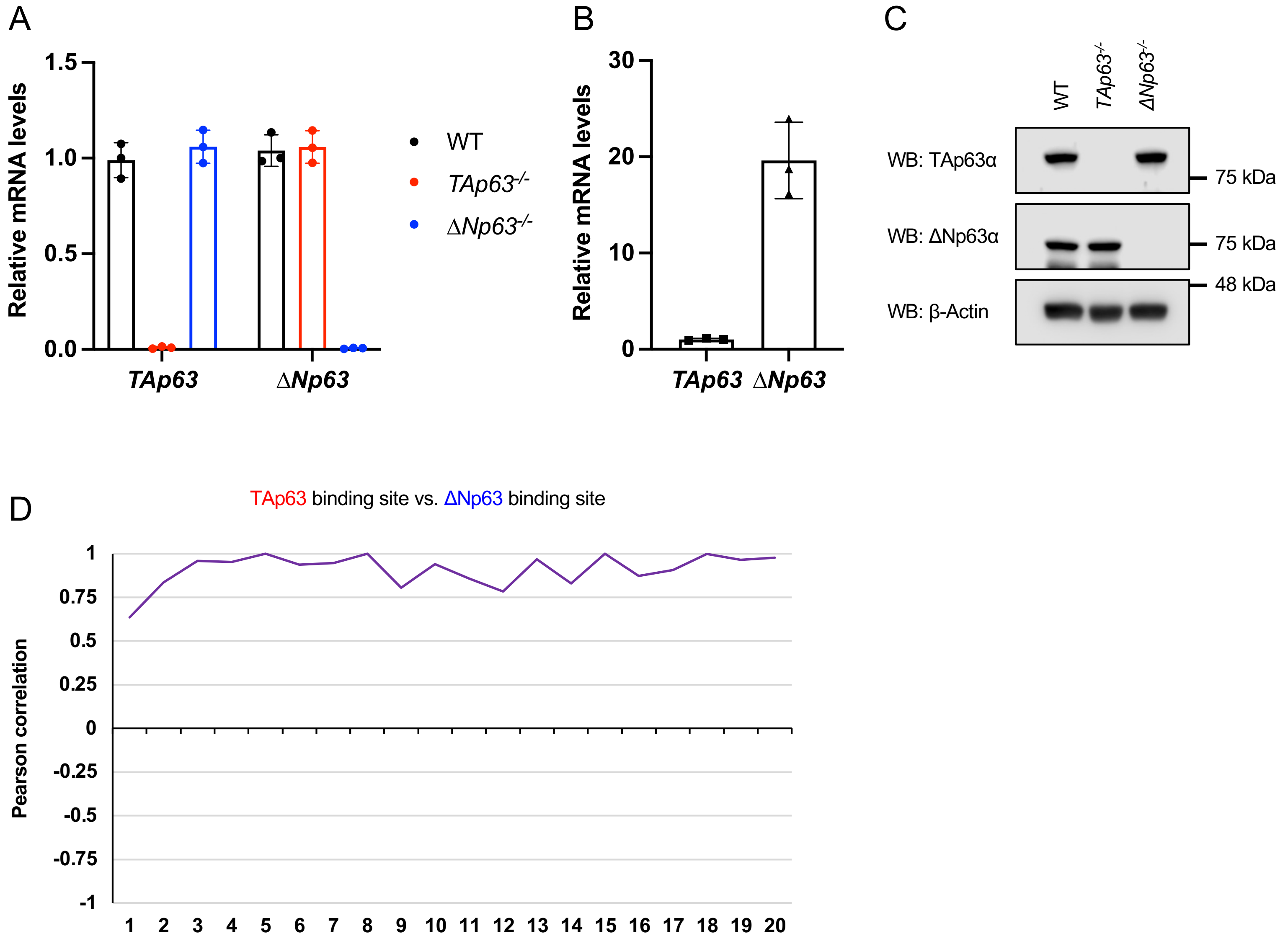

Fig. S1.
Endogenous ΔNp63 and TAp63 bind to distinct regions throughout the genome.
A, qRT-PCR for *TAp63* and *ΔNp63* in epidermal cells of the indicated genotype. **B,** Relative mRNA levels of p63 isoforms. **C,** Representative western blot analysis using the indicated antibodies of the endogenous ΔNp63 and TAp63 in epidermal cells of the indicated genotype. **D,** Pearson correlation analysis of the p63 isoform-specific response elements at nucleotide level.
