# Supplementary material for: Genome-wide p63-Target Gene Analyses Reveal TAp63/NRF2-Dependent Oxidative Stress Responses
Source: Cancer Res Commun. 2024 Feb 1;4(2):264–78. doi: 10.1158/2767-9764.CRC-23-0358 (PMC10832605; doi:10.1158/2767-9764.CRC-23-0358)
Supplement: Supplementary Figure S2 — Identification of ∆Np63 and TAp63 regulated transcriptomes [file crc-23-0358-s02.pdf]

Supplementary Figure 2

A

	Number of differentially expressed genes				NES scores			
	ΔNp63		TAp63		Rank file ΔNp63 regulated genes vs. TAp63 signatures		Rank file TAp63 regulated genes vs. ΔNp63 signatures	
	UP genes	DOWN genes	UP genes	DOWN genes	UP genes	DOWN genes	UP genes	DOWN genes
FC 1.25x, FDR<0.05	4569	4577	1259	1348	14.50	-20.99	20.74	-26.36
FC 2x, FDR<0.05	2694	2704	614	625	12.10	-17.29	17.94	-24.11
FC 4x, FDR<0.05	1541	1649	120	180	6.36	-10.69	15.38	-21.08
FC 8x, FDR<0.05	1035	1231	32	53	3.90	-6.15	13.37	-18.86

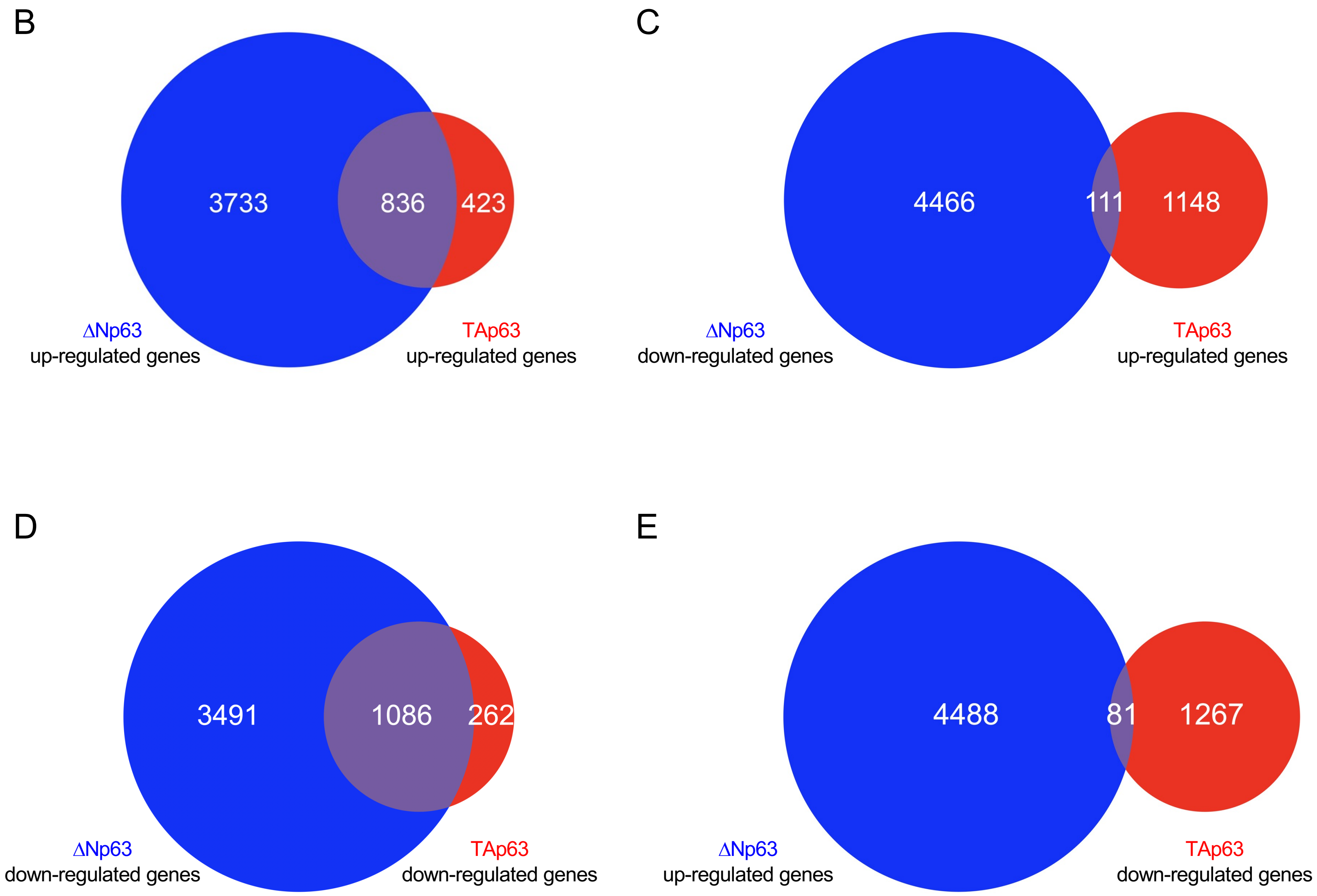

Fig. S2.
Identification of ΔNp63 and TAp63 regulated transcriptomes.
A, Table listing the number of genes regulated by the p63 isoforms at the indicated fold change cut-offs and the NES scores for the relative GSEA analysis. **B-E,** Venn diagrams of the genes upregulated or downregulated by ΔNp63 or TAp63 in epidermal cells.
