# Supplementary material for: Genome-wide p63-Target Gene Analyses Reveal TAp63/NRF2-Dependent Oxidative Stress Responses
Source: Cancer Res Commun. 2024 Feb 1;4(2):264–78. doi: 10.1158/2767-9764.CRC-23-0358 (PMC10832605; doi:10.1158/2767-9764.CRC-23-0358)
Supplement: Supplementary Figure S3 — ∆Np63 cooperates with the FOX family members to regulate cell motility genes [file crc-23-0358-s03.pdf]

Supplementary Figure 3

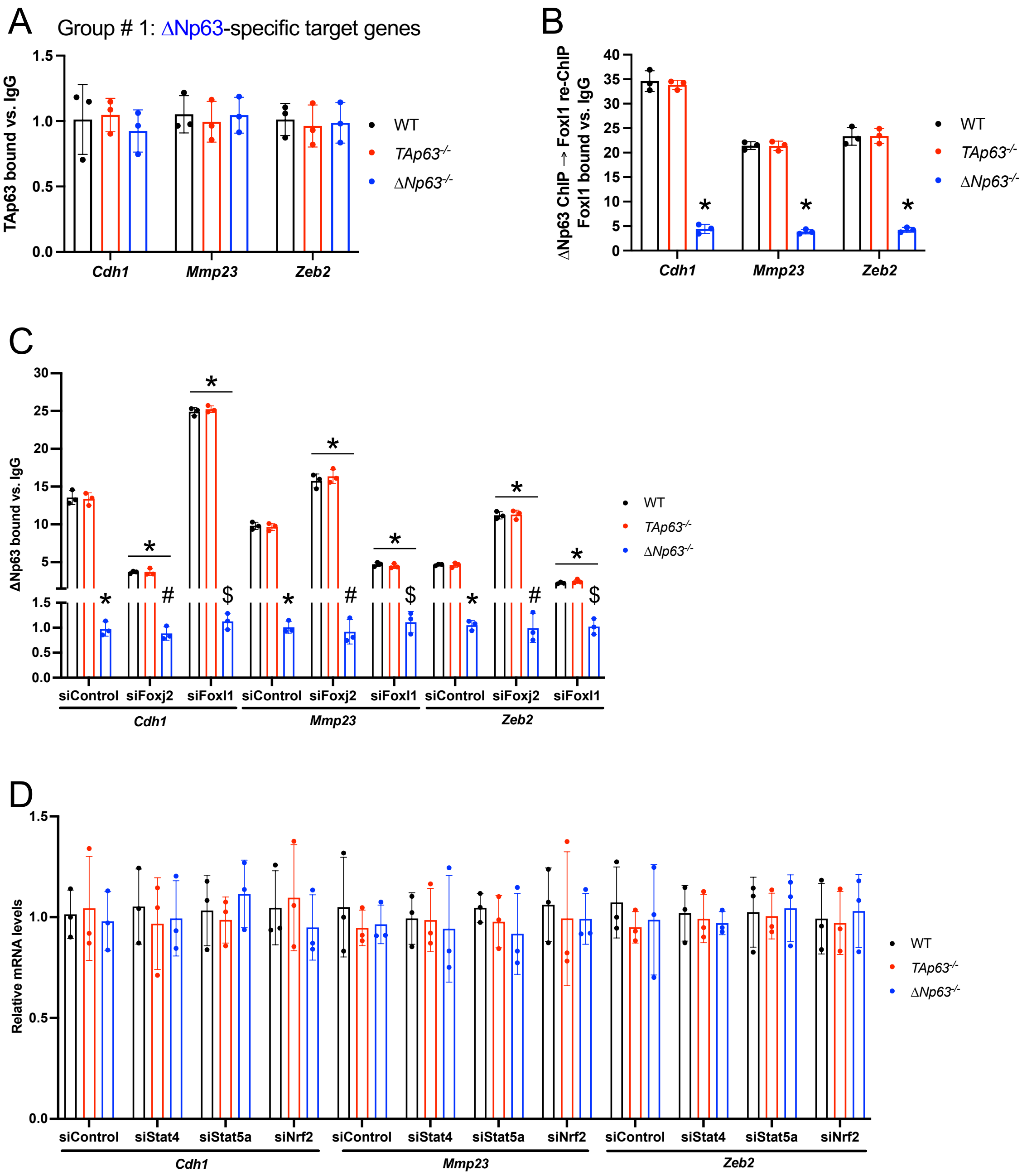

Fig. S3.

ΔNp63 cooperates with the FOX family members to regulate cell motility genes.

A, qRT-PCR of TAp63 ChIP assays using epidermal cells of the indicated genotype on the ΔNp63-specific peaks of the indicated ΔNp63-specific target genes. Data are mean ± SD, n = 3. **B,** qRT-PCR of Foxl1 ChIP-re-ChIP assays on the ΔNp63 ChIP-ed genomic regions of the indicated ΔNp63-specific target genes using WT, $\Delta Np63^{-/-}$, and $TAp63^{-/-}$ epidermal cells. Data are mean ± SD, n = 3. * vs. WT, $P < 0.005$, two-tailed t-test. **C,** qRT-PCR of ΔNp63 ChIP assay on the ΔNp63-specific peaks of the indicated ΔNp63-specific target genes using WT, $\Delta Np63^{-/-}$, and $TAp63^{-/-}$ epidermal cells transfected with the indicated siRNAs. Data are mean ± SD, n = 3, * vs. WT siControl, **#** vs. WT siFoxj2, and $ vs. WT siFoxl1, $P < 0.005$, two-tailed t-test. **D,** qRT-PCR of the indicated ΔNp63-specific target genes in WT, $\Delta Np63^{-/-}$, and $TAp63^{-/-}$ epidermal cells transfected with the indicated siRNAs. Data are mean ± SD, n = 3.
