# Supplementary material for: Genome-wide p63-Target Gene Analyses Reveal TAp63/NRF2-Dependent Oxidative Stress Responses
Source: Cancer Res Commun. 2024 Feb 1;4(2):264–78. doi: 10.1158/2767-9764.CRC-23-0358 (PMC10832605; doi:10.1158/2767-9764.CRC-23-0358)
Supplement: Supplementary Figure S4 — ∆Np63 and TAp63 control the expression of their common target genes together with Stat proteins [file crc-23-0358-s04.pdf]

Supplementary Figure 4

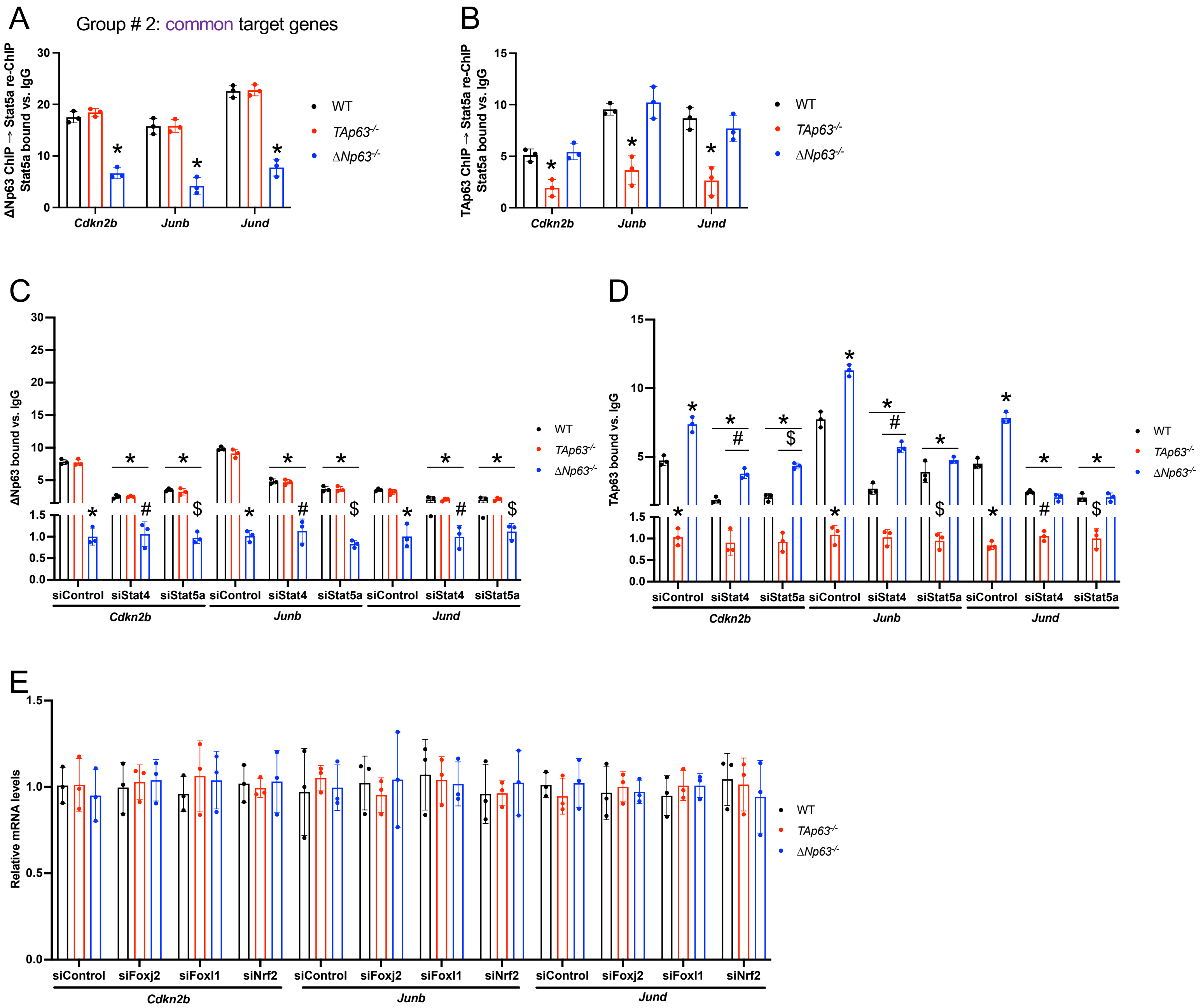

Fig. S4.

ΔNp63 and TAp63 control the expression of their common target genes together with Stat proteins.

A, qRT-PCR of Stat5a ChIP-re-ChIP assays using the ΔNp63 ChIP-ed genomic regions of the indicated common target genes from epidermal cells of the indicated genotype. Data are mean ± SD, n = 3, * vs. WT, $P < 0.005$, two-tailed t-test. **B,** qRT-PCR of Stat5a ChIP-re-ChIP assays using the TAp63 ChIP-ed genomic regions of the indicated common target genes from epidermal cells of the indicated genotype. Data are mean ± SD, n = 3, * vs. WT, $P < 0.005$, two-tailed t-test. **C,** qRT-PCR of ΔNp63 ChIP assay on the common peaks of the indicated common target genes using WT, *ΔNp63*$^{-/-}$, and *TAp63*$^{-/-}$ epidermal cells transfected with the indicated siRNAs. Data are mean ± SD, n = 3, * vs. WT siControl, # vs. WT siStat4, and $ vs. WT siStat5a, $P < 0.005$, two-tailed t-test. **D,** qRT-PCR of TAp63 ChIP assay on the same samples as in C. Data are mean ± SD, n = 3, * vs. WT siControl, # vs. WT siStat4, and $ vs. WT siStat5a, $P < 0.005$, two-tailed t-test. **E,** qRT-PCR of the indicated common target genes in WT, *ΔNp63*$^{-/-}$, and *TAp63*$^{-/-}$ epidermal cells transfected with the indicated siRNAs. Data are mean ± SD, n = 3.
