# Supplementary material for: Genome-wide p63-Target Gene Analyses Reveal TAp63/NRF2-Dependent Oxidative Stress Responses
Source: Cancer Res Commun. 2024 Feb 1;4(2):264–78. doi: 10.1158/2767-9764.CRC-23-0358 (PMC10832605; doi:10.1158/2767-9764.CRC-23-0358)
Supplement: Supplementary Figure S5 — TAp63 and NRF2 coordinately regulate the expression of TAp63-specific target genes [file crc-23-0358-s05.pdf]

Supplementary Figure 5

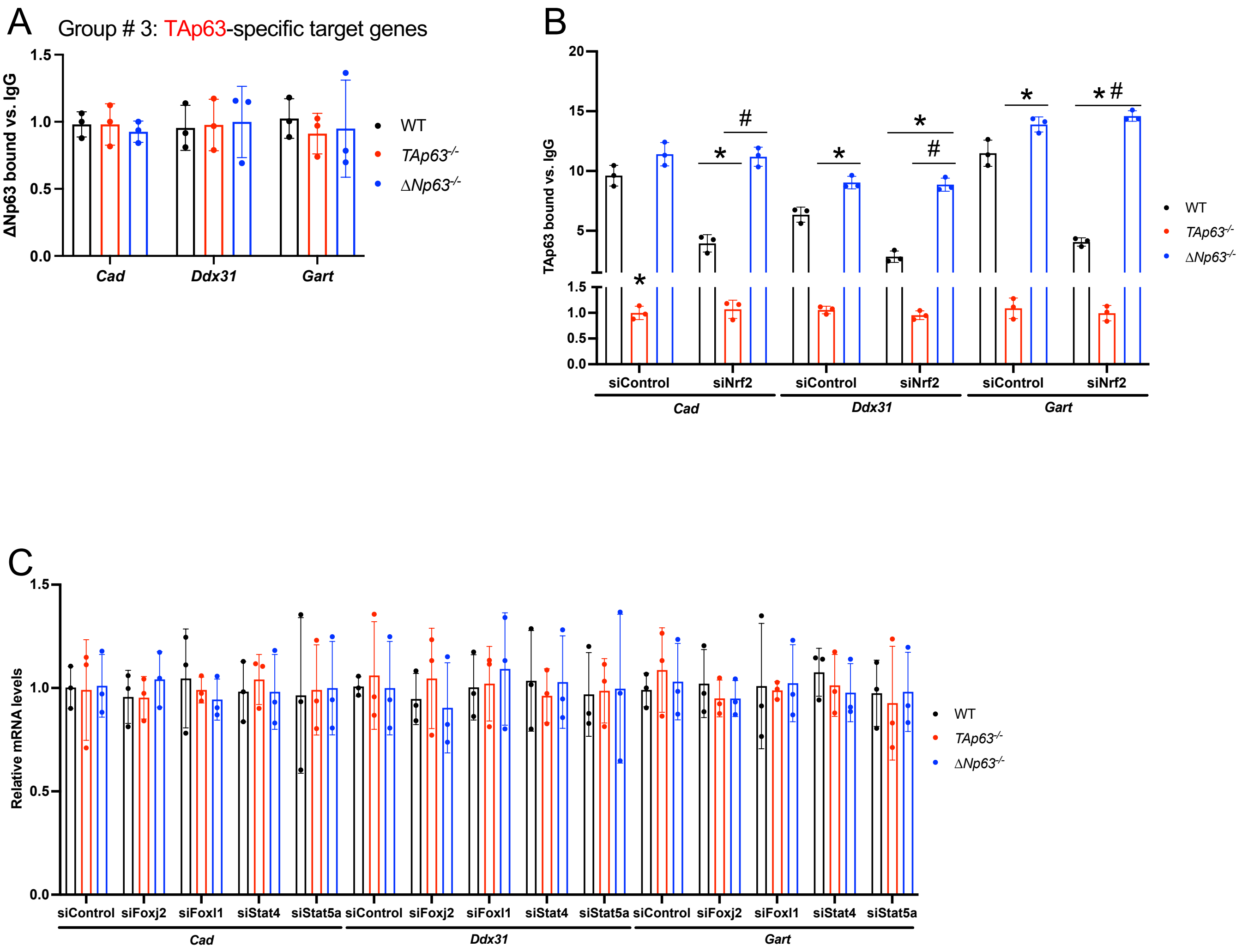

Fig. S5.

TAp63 and NRF2 coordinately regulate the expression of TAp63-specific target genes.

A, qRT-PCR of ΔNp63 ChIP assays using epidermal cells of the indicated genotype on the TAp63-specific peaks of the indicated TAp63-specific target genes. Data are mean ± SD, n = 3. **B,** qRT-PCR of TAp63 ChIP assay on the TAp63-specific peaks of the indicated TAp63-specific target genes using WT, *ΔNp63*$^{-/-}$, and *TAp63*$^{-/-}$ epidermal cells transfected with the indicated siRNAs. Data are mean ± SD, n = 3, * vs. WT siControl, # vs. WT siNrf2, $P < 0.005$, two-tailed t-test. **C,** qRT-PCR of the indicated TAp63-specific target genes in WT, *ΔNp63*$^{-/-}$, and *TAp63*$^{-/-}$ epidermal cells transfected with the indicated siRNAs. Data are mean ± SD, n = 3.
