# Supplementary material for: Genome-wide p63-Target Gene Analyses Reveal TAp63/NRF2-Dependent Oxidative Stress Responses
Source: Cancer Res Commun. 2024 Feb 1;4(2):264–78. doi: 10.1158/2767-9764.CRC-23-0358 (PMC10832605; doi:10.1158/2767-9764.CRC-23-0358)
Supplement: Supplementary Figure S6 — TAp63 promotes the NRF2-dependent oxidative stress response [file crc-23-0358-s06.pdf]

Supplementary Figure 6

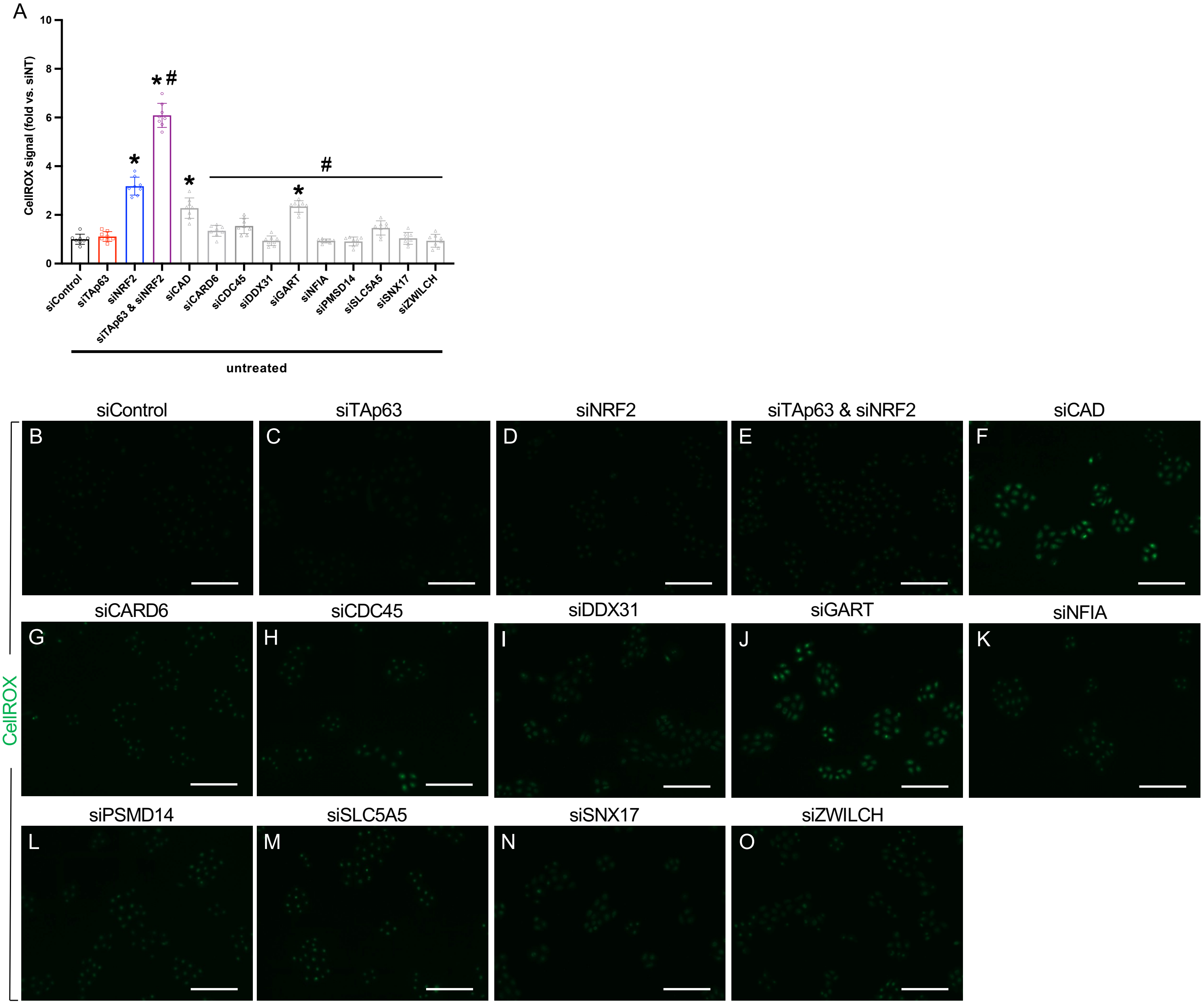

Fig. S6.

TAp63 promotes the NRF2-dependent oxidative stress response.

A, Quantification of the CellRox signal in KER-CT cells transfected with the indicated siRNAs. The CellRox signal is normalized with respect to the siControl cells. Data are mean ± SD, n = 8, * vs. siControl, **#** vs. siNRF2 H_2O_2, $P < 0.005$, two-tailed t-test. **B-O,** Representative images of CellRox signal in KER-CT cells transfected with the indicated siRNAs. Scale bars equal 100µm.
